# Reported adverse events following monovalent varicella vaccination in children aged 1–12 years: a VAERS disproportionality analysis, 1995-2026

**DOI:** 10.3389/fimmu.2026.1959638

**Published:** 2026-10-29

**Authors:** Meishen Liu, Yalin Xi, Le Yang, Miao Li, Depeng Zhao, Tangna Hao, Weijia Sun, Xinkuo Zheng

**Affiliations:** 1 Department of Pharmacy, The Second Affiliated Hospital of Dalian Medical University, Dalian, Liaoning, China; 2 Department of Pharmacy, Central Hospital of Dalian University of Technology, Dalian, Liaoning, China

**Keywords:** children, pharmacovigilance, safety surveillance, VAERS, varicella vaccine

## Abstract

**Background:**

Varicella vaccines have been widely used in children, but comprehensive post-marketing safety evaluations in the pediatric population remain limited.

**Methods:**

We analyzed 55,475 adverse event (AE) reports following varicella vaccination in children aged 1–12 years from the U.S. Vaccine Adverse Event Reporting System (VAERS) between 1995 and 2026. Descriptive analyses were performed for demographic and clinical characteristics. Disproportionality signals were detected using Proportional Reporting Ratio (PRR) and Reporting Odds Ratio (ROR), with false discovery rate controlled by Benjamini-Hochberg (BH) correction. Stratified analyses were conducted by age and sex.

**Results:**

The most frequently reported adverse events were rash (a = 7,218, ROR = 2.09, PRR = 2.05), infection (a = 5,694, ROR = 10.76, PRR = 10.43), dermatitis bullous (a = 3,563, ROR = 37.28, PRR = 36.51), viral infection (a = 2,173, ROR = 9.67, PRR = 9.56) and rash vesicular (a = 2,137, ROR = 20.94, PRR = 20.68). Most cases (53.72%) resolved completely. Children aged 1–2 years accounted for 52.00% of reports. Initial analysis identified 135 positive signals at the Preferred Term level, which were reduced to 111 after multiplicity correction. Injection site reactions predominated in older children (3–6 and 7–12 years), while systemic events were more common in the youngest group. Sex differences were minimal. Serious adverse events occurred in 5.94% and deaths in 0.30% of reports.

**Conclusion:**

This analysis of VAERS reports describes a generally non-serious and self-limiting reporting profile for varicella vaccines in children aged 1–12 years. These findings support continued pharmacovigilance to inform evidence-based vaccination policies and targeted risk communication, but they do not confirm a favorable safety profile or establish causality, incidence, or absolute risk. Further denominator-based studies are needed to validate these observations.

## Introduction

Varicella, commonly known as chickenpox, is an acute and highly contagious disease caused by primary infection with varicella-zoster virus, a neurotropic human alpha-herpesvirus (1, 2). Transmission occurs mainly through respiratory droplets, aerosols from vesicular fluid, and direct contact with skin lesions (3). Before the introduction of routine immunization, varicella was nearly universal in childhood in the United States, with most individuals infected before adolescence (3). Although often regarded as a self-limited illness in otherwise healthy children, varicella can lead to substantial morbidity, including bacterial superinfection of skin lesions, pneumonia, cerebellar ataxia, encephalitis, dehydration, and hospitalization (1). Severe outcomes are more likely in infants, adolescents, adults, pregnant women, and immunocompromised individuals, but complications also occur in healthy pediatric populations (1). In addition to its direct clinical burden, varicella historically imposed considerable societal and economic costs through outpatient visits, hospital admissions, parental work loss, and school absenteeism (4).

The development and implementation of live-attenuated varicella vaccines represented a major advance in pediatric infectious disease prevention (5). Routine childhood vaccination was introduced to reduce the incidence of primary varicella infection and its associated complications (6, 7). Over time, immunization strategies evolved from a single-dose schedule to a two-dose schedule in response to evidence that breakthrough infections could occur after one dose and that a second dose improved protection against disease (8, 9). In the United States, varicella vaccination has been incorporated into routine pediatric immunization programs, typically beginning at 12 through 15 months of age, with a second dose administered at 4 through 6 years of age (10). High vaccine coverage has been associated with marked declines in varicella incidence, outbreaks, hospitalizations, and deaths, demonstrating substantial public health benefit across pediatric age groups (11).

The currently used varicella vaccines are live-attenuated preparations derived from the Oka strain of varicella-zoster virus (5). These vaccines may be administered as monovalent varicella vaccine or as part of combination formulations such as measles-mumps-rubella-varicella vaccine (9). Because they are live vaccines, their use requires attention to specific contraindications and precautions, particularly in children with severe immunodeficiency, certain malignancies, or known severe allergic reactions to vaccine components (10). In immunocompetent children, however, varicella vaccines have generally demonstrated favorable safety and immunogenicity profiles in both pre-licensure trials and post-licensure evaluations (5). Most adverse events (AEs) reported after vaccination are mild and self-limited, including injection-site reactions, fever, and rash (12). Nonetheless, rare but clinically important events, such as febrile seizures, allergic reactions, thrombocytopenia, or vaccine-strain varicella-related manifestations, remain important to monitor in large populations after widespread use (13, 14).

Post-marketing safety surveillance is particularly important for pediatric vaccines because rare AEs may not be fully detectable in pre-licensure studies, which are often limited by sample size, duration of follow-up, and selective study populations (15). Passive surveillance systems such as the U.S. Vaccine Adverse Event Reporting System (VAERS) provide an important mechanism for detecting unusual or unexpected safety signals, characterizing reporting patterns, and generating hypotheses for further evaluation (16). Although such systems cannot establish causality and are subject to underreporting, stimulated reporting, incomplete clinical detail, and reporting bias, they remain valuable tools for identifying potential concerns that warrant more rigorous epidemiologic investigation (16).

In the context of varicella vaccination, post-marketing surveillance is particularly relevant because the target population is young children, many of whom receive multiple vaccines during the same visit (15). This co-administration pattern can complicate attribution of AEs and underscores the importance of careful signal assessment (13). Furthermore, the distinction between vaccine-related reactions, coincidental illnesses, and manifestations of wild-type varicella infection may be challenging in routine reporting data (17). Evaluating safety specifically among children aged 1 to 12 years is clinically meaningful because this age range encompasses the primary period of vaccine administration, includes both first-dose and second-dose recipients, and captures the population in which the public health impact of vaccination is greatest (18).

Despite the well-established effectiveness of varicella vaccination, continued assessment of safety remains essential for maintaining public confidence in immunization programs (15). Since the introduction of routine varicella vaccination in the United States in 1995, an estimated 110–140 million doses of varicella vaccine have been administered to children aged 1–12 years. This estimate is based on U.S. birth cohort data from the National Vital Statistics System and national vaccination coverage estimates from the National Immunization Survey-Child (19). Coverage with ≥1 dose of varicella vaccine increased from 12.0% in 1996 to 89.2% by 2006 and has remained at or above 90% since. Following the 2007 recommendation for a routine 2-dose schedule, 2-dose coverage among children aged 7 years increased from 2.6%-5.5% in 2006 to 86.0%-100.0% by 2020 (20). However, pediatric-specific VAERS disproportionality analyses focusing on monovalent varicella vaccines over a long study period remain limited. To address this gap, we examined reported AEs following monovalent varicella vaccination in children aged 1 to 12 years using U.S. VAERS data from 1995 to 2026. By describing demographic and clinical characteristics, summarizing AE types, and identifying positive AE signals, this analysis aims to provide updated pediatric pharmacovigilance evidence and a basis for future analytic studies.

## Materials and methods

### Data source

The VAERS database, a national surveillance system of the United States under the joint administration of the CDC and the Food and Drug Administration (FDA), served as the data source for this investigation (21). Since its inception in 1990, VAERS has constituted a fundamental element of the post-licensure vaccine safety monitoring framework within the U.S. pharmacovigilance system. Its principal functions encompass the identification of novel, unusual, or infrequent AEs, the surveillance of trends in known AEs, the detection of patient-level risk determinants, and the safety assessment of vaccines that have recently received licensure (22). Within this system, AE manifestations are encoded using standardized MedDRA (version 27.1) Preferred Term (PT) codes, and each VAERS case report accommodates documentation of as many as five distinct clinical presentations. To ensure consistency across the study period, all reported terms were standardized to MedDRA v27.1. Historical terms were mapped using MSSO version-to-version mapping files, and where only PT text was available, exact matching against the MedDRA v27.1 dictionary was performed, followed by manual review. The assignment of PTs to system organ classes (SOCs) is executed as an integral component of the analytical workflow (23).

For the purposes of the current study, VAERS reports pertaining to varicella vaccines were systematically examined over the interval from January 1, 1995, through June 30, 2026. The extracted dataset encompassed all reports corresponding to the following vaccine formulations: VARICELLA (VARIVAX), VARICELLA (VARILRIX), and VARICELLA (NO BRAND NAME). Only monovalent varicella vaccine records were included; combination vaccines containing varicella antigen, such as MMRV/ProQuad, were not included. However, reports that contained a monovalent varicella vaccine record together with other non-varicella vaccines were still retained, because the exposure definition was based on the presence of at least one eligible monovalent varicella vaccine record. To ensure comprehensive capture of all reported AEs, the analytical process imposed no geographic or clinical restrictions. Duplicate entries were exceedingly scarce, and where present, they were removed through a deduplication procedure. With respect to demographic variables, we deliberately restricted the sample to children between 1 and 12 years of age who had received varicella vaccination, to specifically evaluate the safety profile in this pediatric cohort, while no constraints were placed on other demographic attributes. In parallel, age-stratified (1–2 years, 3–6 years, and 7–12 years) and sex-stratified subgroup analyses were performed to facilitate a more granular assessment of varicella vaccine safety. Serious adverse events (SAEs) and death outcomes were likewise subjected to separate analytical evaluation. SAEs were defined according to FDA 21 CFR 600.80 and ICH E2A criteria as events that resulted in death, were life-threatening, required hospitalization or prolongation of hospitalization, caused persistent or significant disability/incapacity, resulted in congenital anomaly/birth defect, or required intervention to prevent permanent impairment or damage. Because these categories are not mutually exclusive, a report was classified as serious if it met at least one criterion, and the total number of SAEs represents unique reports with at least one serious outcome. Individual serious outcome categories may overlap, and their frequencies do not sum to the total number of serious reports.

### Descriptive analysis

A descriptive characterization of varicella related AEs was undertaken based on the collected reports, with data disaggregated according to sex, age, primary clinical outcome categories (including death, disability, hospitalization, life threatening events, extended hospitalization, and recovery), and time from vaccination to symptom onset. Additionally, vaccines were classified by product designation, manufacturer, and dose number, and the annual report counts from 1995 through June 2026 were examined for each of these classification strata.

### Statistical analysis

Our pharmacovigilance approach incorporated two disproportionality assessment techniques: the Proportional Reporting Ratio (PRR) (24) and the Reporting Odds Ratio (ROR) (25). Each of these algorithms offers distinct benefits; PRR is characterized by high specificity, whereas ROR exhibits superior performance relative to PRR in mitigating reporting biases that affect infrequent events. The combined application of these complementary strategies serves to offset the shortcomings inherent in each isolated method through mutual cross-validation. With the objective of reducing the likelihood that a true signal would be overlooked, a positive signal was declared only when the criteria from both methods were concurrently satisfied. A standardized 2×2 contingency table format (**Supplementary Table S1**) was uniformly adopted, with consistent computational formulae and prespecified statistical thresholds (**Supplementary Table S2**) applied throughout. A greater magnitude of the signal strength was interpreted as indicating a higher probability of a genuine association between the vaccine and the AE under consideration. After the initial signal identification, the Benjamini-Hochberg (BH) procedure was implemented on the χ^2^ test results derived from the PRR analysis to regulate the false discovery rate (FDR) and to curtail the elevation of Type I error arising from multiple hypothesis testing. A potential AE signal was deemed statistically significant only when its BH-adjusted *p*-value fell below the predefined FDR threshold of Q = 0.05. Vaccine-event pairs that satisfied this criterion were designated as exhibiting positive disproportionality signals. All statistical computations were carried out using R software (version 4.4.2) and Microsoft Excel 2021.

## Results

### Descriptive characteristics of reported AEs

This investigation included 55,475 recipients of varicella vaccination aged 1–12 years (**Table 1**). Stratification by sex showed a slightly lower incidence of AEs among female participants, who accounted for 46.51% of cases, compared with male participants, who accounted for 47.79%. Age-stratified subgroup analysis demonstrated that the highest proportion of affected individuals was observed in the youngest subgroup, namely children aged 1–2 years, who comprised 52.00% of all reported AEs. Among individuals who experienced AEs following varicella vaccination, excluding cases with undocumented clinical outcomes, complete recovery was the most frequently observed outcome, occurring in 53.72% of patients. Hospitalization attributable to the reported AEs was documented in 4.58% of cases, representing the second most common clinical outcome in this cohort. Temporal assessment revealed that 52.39% of patients experienced AEs within 0–6 days after vaccination. Within this time window, 22,188 cases had an onset time of ≤1 day, accounting for approximately 40.00% of all AEs. With respect to reporting year, the highest number of reports was observed in 2008, with 4,429 cases accounting for 7.98% of the total (**Figure 1A**). Regarding reporting region, California had the highest number of reports, with 4,850 cases representing 8.74% of all reports (**Figure 1B**).

**Table 1:** Characteristics of AE reports associated with varicella vaccines from VEARS between 1995 and 2026.

Characteristics	Number (%)
Total	55,475 (100%)
Sex	
Female	25,801 (46.51%)
Male	26,514 (47.79%)
N/A	3,160 (5.70%)
Age (year)	
≥ 1 and ≤ 2	28,849 (52.00%)
≥ 3 and ≤ 6	16,925 (30.51%)
≥ 7 and ≤ 12	9,701 (17.49%)
Primary clinical outcome	
Died	165 (0.30%)
Disability	555 (1.00%)
Hospitalized	2,538 (4.58%)
Life threatening	605 (1.09%)
Prolonged hospitalization	133 (0.24%)
Recovered	29,808 (53.72%)
Onset time (day)	
≥ 0 and ≤ 6	29,064 (52.39%)
≥ 7 and ≤ 13	7,316 (13.19%)
≥ 14 and ≤ 20	1,819 (3.28%)
≥ 21 and ≤ 28	694 (1.25%)
≥ 29	1,1033 (19.89%)
N/A	5,549 (10.00%)

AE, adverse event; VAERS, vaccine adverse event reporting system; N/A, not available.

**Figure 1:**
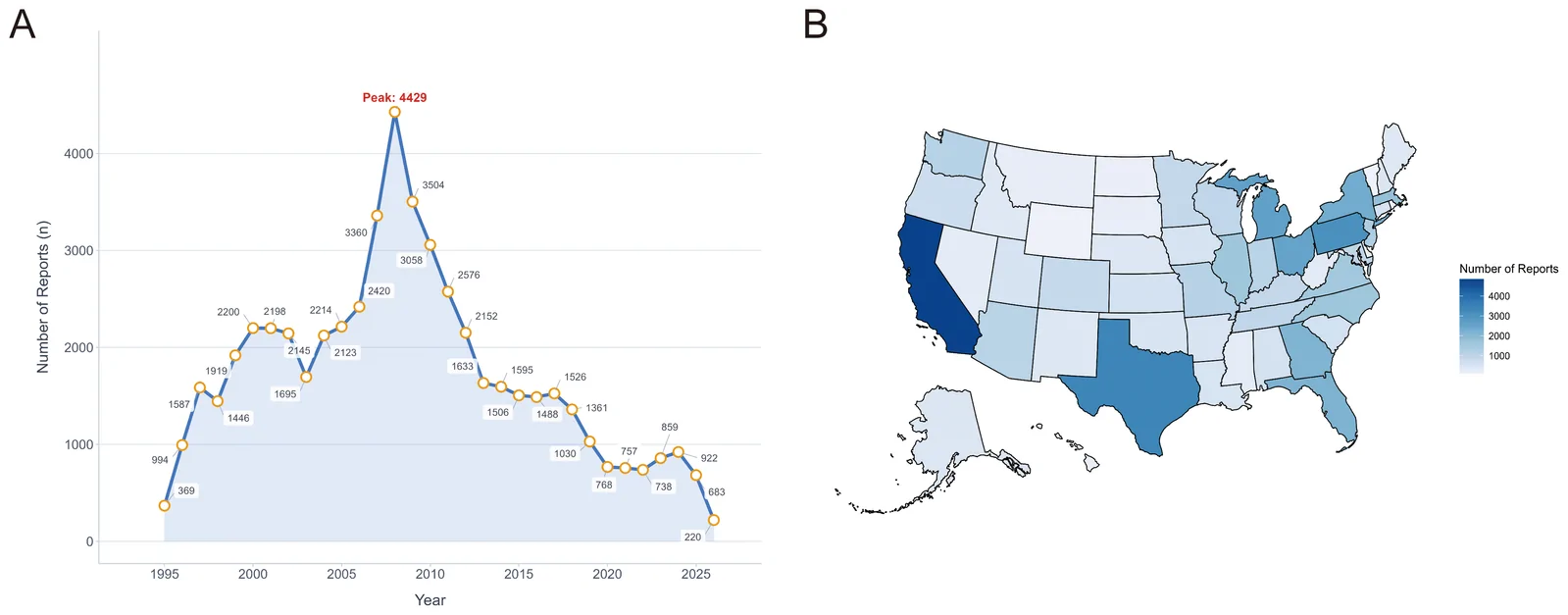
Temporal **(A)** and geographic **(B)** distribution of reported case counts for varicella vaccine-related adverse events.

A total of 55,478 varicella vaccine records associated with the included VAERS reports were identified. This number exceeded the total number of AE reports (n = 55,475) by three because three vaccine recipients received two different varicella vaccine products. These three recipients therefore contributed three additional vaccine records. Among all records, Varivax was the predominant vaccine formulation, accounting for 95.88% of all doses. Regarding manufacturer, Merck & Co., Inc. supplied nearly all vaccines (95.88%), whereas GlaxoSmithKline Biologicals contributed only a small proportion (0.41%). When categorized by dose number within the immunization schedule, first dose records were the most frequently reported among the included VAERS records, accounting for 49.59%, followed by the second dose at 24.00% (**Table 2**). The annual distribution of reported AEs across calendar years is shown in **Figure 1**.

**Table 2:** Characteristics of varicella vaccine records associated with included VAERS reports between 1995 and 2026.

Characteristics	Number (%)
Total vaccines	55,478 (100%)
Vaccine name	
Varivax	53,195 (95.88%)
Varilrix	229 (0.41%)
No brand name	2,054 (3.70%)
Vaccine manufacturer	
Merck & Co. Inc.	53,195 (95.88%)
GlaxoSmithKline Biologicals	229 (0.41%)
N/A	2,054 (3.70%)
Administration dose	
1	27,509 (49.59%)
2	13,312 (24.00%)
3	541 (0.98%)
4	68 (0.12%)
5	21 (0.04%)
6	9 (0.02%)
7+	16 (0.03%)
N/A	14,002 (25.24%)

VAERS, vaccine adverse event reporting system; N/A, not available.

### Disproportionality analysis

An examination of VAERS data from children aged 1 to 12 years revealed 3,316 varicella vaccine-related PTs, which were distributed across 27 SOCs. When two pharmacovigilance methods were applied, the assessment generated 135 positive PT signals, and these signals involved 18 SOCs. In terms of the positive AE signals, the five PTs with the greatest occurrence frequencies were rash (a = 7,218, ROR = 2.09, PRR = 2.05), infection (a = 5,694, ROR = 10.76, PRR = 10.43), dermatitis bullous (a = 3,563, ROR = 37.28, PRR = 36.51), viral infection (a = 2,173, ROR = 9.67, PRR = 9.56) and rash vesicular (a = 2,137, ROR = 20.94, PRR = 20.68). As shown in **Figure 2**, the top 20 positive PT signals for varicella vaccines are presented, and **Supplementary Table S3** provides a complete list of all positive PT signals that were identified.

**Figure 2:**
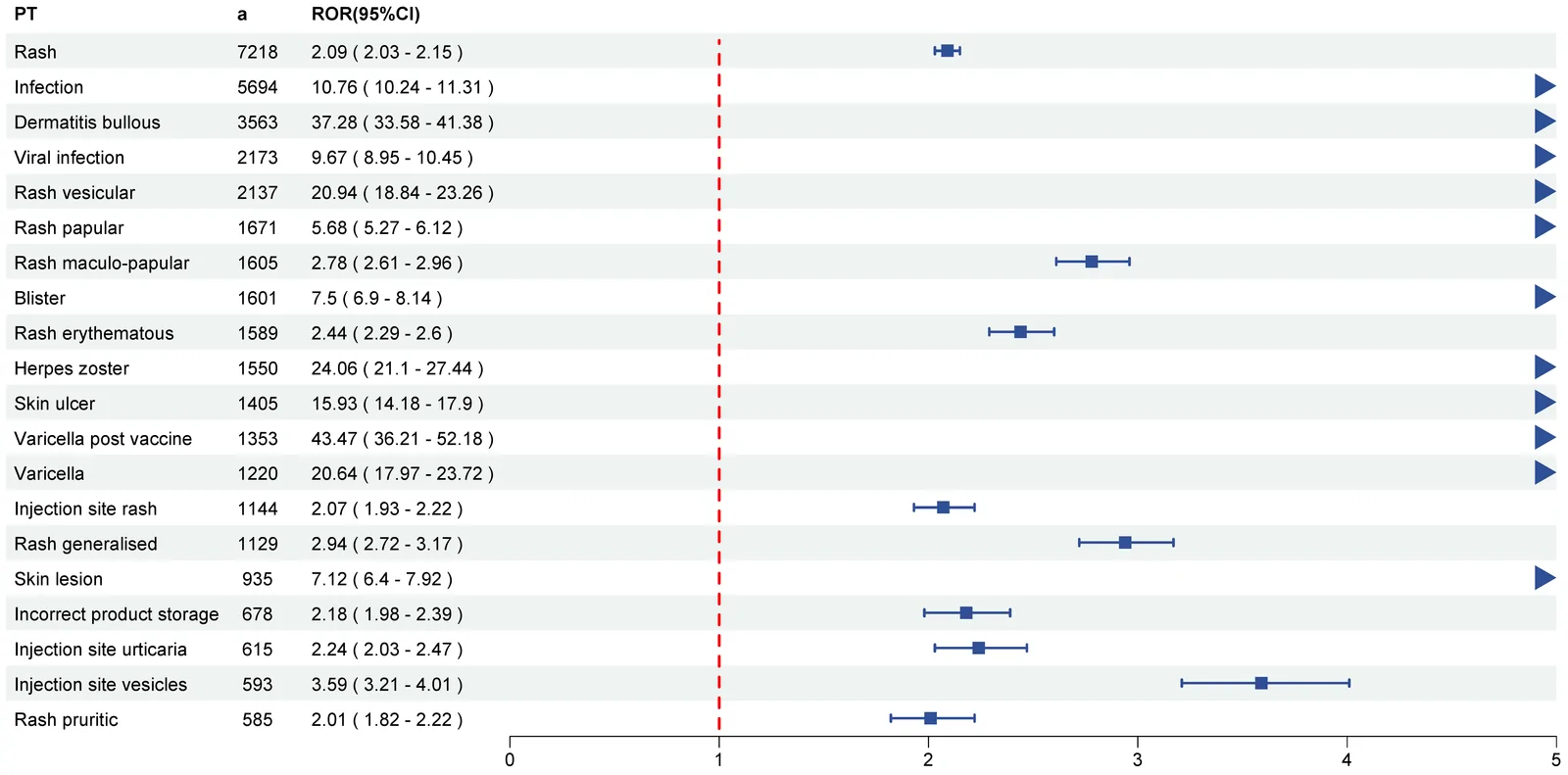
The forest plot of top twenty varicella vaccine-related positive PT signals. PT, preferred term; ROR, reporting odds ratio.

After BH correction was applied to the 135 positive PT signals (**Supplementary Table S3**), 23 of them were found to have *p*-adjust values that no longer met the significance criterion, thus they were reclassified as nonpositive signals. These 24 PTs comprised ear tube insertion (a = 10, *p*-adjust = 0.055), exfoliative rash (a = 9, *p*-adjust = 0.065), herpes simplex test (a = 8, *p*-adjust = 0.056), blood lead normal (a = 7, *p*-adjust = 0.066), cd4 lymphocytes decreased (a = 7, *p*-adjust = 0.066), malabsorption (a = 6, *p*-adjust = 0.077), aspiration bone marrow abnormal (a = 6, *p*-adjust = 0.052), culture wound positive (a = 6, *p*-adjust = 0.077), abscess bacterial (a = 5, *p*-adjust = 0.087), lip ulceration (a = 5, *p*-adjust = 0.087), bone marrow failure (a = 5, *p*-adjust = 0.087), herpes simplex serology positive (a = 5, *p*-adjust = 0.087), cd8 lymphocytes decreased (a = 4, *p*-adjust = 0.055), fluctuance (a = 4, *p*-adjust = 0.055), eschar (a = 4, *p*-adjust = 0.055), septic screen (a = 3, *p*-adjust = 0.092), cell marker (a = 3, *p*-adjust = 0.092), diabetes mellitus insulin-dependent (a = 3, *p*-adjust = 0.092), herpes zoster disseminated (a = 3, *p*-adjust = 0.092), onychomadesis (a = 3, *p*-adjust = 0.092), tuberculin test positive (a = 3, *p*-adjust = 0.092), supportive care (a = 3, *p*-adjust = 0.092), and respiratory tract oedema (a = 3, *p*-adjust = 0.092). Following BH correction, the remaining 111 PTs continued to be classified as positive signals.

Among the SOCs assessed, the five with the highest reporting frequencies were general disorders and administration site conditions (a = 59,651, ROR = 1.10, PRR = 1.07), skin and subcutaneous tissue disorders (a = 38,744, ROR = 2.47, PRR = 2.13), infections and infestations (a = 17,298, ROR = 2.69, PRR = 2.52), investigations (a = 12,399, ROR = 0.68, PRR = 0.70), and nervous system disorders (a = 9,340, ROR = 0.50, PRR = 0.53). The relationship between ROR-based signal strength and statistical significance for SOC-level AEs occurring after varicella vaccination is illustrated in **Figure 3**. Full details of the signal strengths for varicella vaccine-related AEs at the SOC level are summarized in **Supplementary Table S4**.

**Figure 3:**
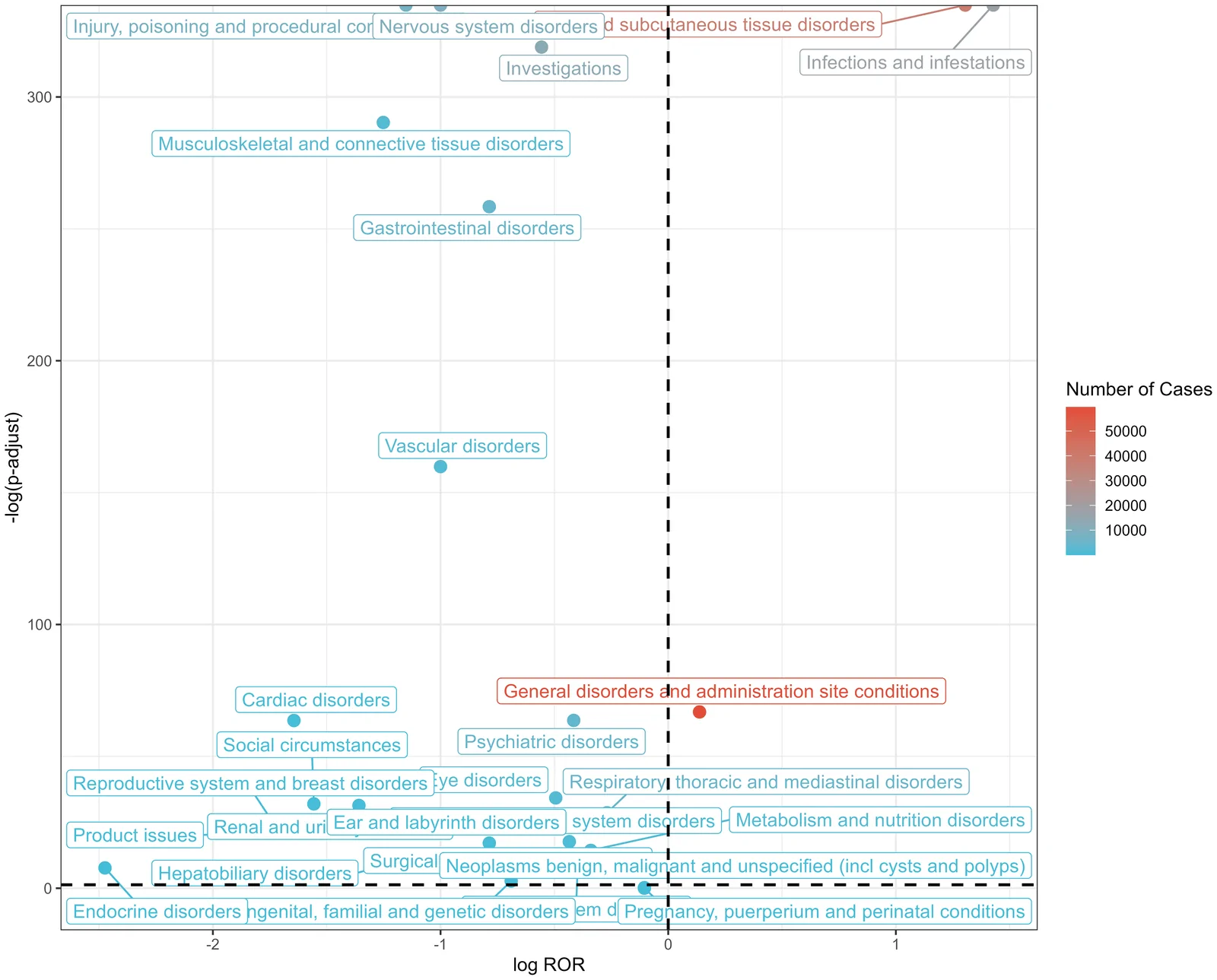
Volcano plot of SOCs corresponding to all reported AEs. SOC, system organ classes; AEs, adverse events; ROR, reporting odds ratio.

### Subgroup analysis

In the context of pharmacovigilance, the application of subgroup analysis serves to diminish the confounding effects stemming from demographic attributes, and it furnishes a multi-faceted outlook for drug safety assessment.

#### PTs across sex groups

To assess the role of sex in the occurrence of side effects from varicella vaccination, we conducted subgroup analyses stratified by sex. **Supplementary Table S5** presents the top ten PTs that met the criteria of both statistical tests in male and female patients.

The top ten significant PTs for the varicella vaccine were consistent between female and male recipients, with only minor differences in the order of a few PTs, such as pruritus and urticaria. Among all reported AEs, pyrexia, which falls under the SOC of general disorders and administration site conditions, accounted for the highest number of reports in both sexes (females: a = 4,991, ROR = 1.18, PRR = 1.17; males: a = 5,215, ROR = 1.07, PRR = 1.07). In addition, injection site reactions were also very frequently reported PTs for the varicella vaccine in men and women. The most common injection site related PTs included injection site erythema (females: a = 4,337, ROR = 1.68, PRR = 1.64; males: a = 3,820, ROR = 1.23, PRR = 1.22), injection site swelling (females: a = 2,581, ROR = 1.64, PRR = 1.62; males: a = 2,473, ROR = 1.25, PRR = 1.24), and injection site warmth (females: a = 1,634, ROR = 1.58, PRR = 1.57; males: a = 1,456, ROR = 1.14, PRR = 1.14). **Supplementary Figure S1** separately illustrates the relationship between signal strength, as measured by the reporting odds ratio (ROR), and statistical significance for SOC level adverse events associated with varicella vaccination in females and males.

#### PTs across age groups

Stratification by age was performed, yielding three subgroups: 1 to 2 years, 3 to 6 years, and 7 to 12 years. Across these three age strata, **Table 3** reports the top ten PTs that satisfied the signal detection thresholds according to both statistical methods concurrently.

**Table 3:** Signal strength of reports of varicella vaccines at the PT level grouped by age.

Age	PT	a	ROR (95%CI)	PRR (χ^2^)
≥ 1 and < 3	Pyrexia	7383	0.93 (0.90-0.95)	0.93 (29.95)
	Rash	5146	1.90 (1.83-1.97)	1.84 (1204.36)
	Infection	2767	5.57 (5.21-5.95)	5.43 (3253.73)
	Injection site erythema	2342	1.09 (1.03-1.14)	1.08 (10.90)
	Dermatitis bullous	1888	26.21 (22.57-30.45)	25.67 (4100.45)
	Erythema	1522	1.47 (1.38-1.56)	1.46 (141.75)
	Urticaria	1480	1.00 (0.94-1.07)	1.00 (0.01)
	Rash vesicular	1319	16.14 (13.96-18.66)	15.91 (2575.59)
	Viral infection	1286	6.16 (5.57-6.81)	6.09 (1632.26)
	Irritability	1273	1.36 (1.27-1.46)	1.36 (79.52)
≥ 3 and < 7	Injection site erythema	3836	1.23 (1.19-1.28)	1.21 (119.15)
	Injection site swelling	2644	1.44 (1.37-1.5)	1.41 (249.23)
	Infection	2062	15.93 (14.52-17.48)	15.32 (6086.17)
	Pyrexia	2057	0.81 (0.77-0.85)	0.81 (77.09)
	Injection site warmth	1723	1.30 (1.23-1.37)	1.29 (88.81)
	Erythema	1484	0.97 (0.91-1.03)	0.97 (1.21)
	Rash	1408	1.61 (1.52-1.72)	1.60 (232.26)
	Dermatitis bullous	1263	38.47 (32.37-45.73)	37.53 (4620.59)
	Injection site pain	1119	0.97 (0.91-1.04)	0.97 (0.76)
	Pruritus	1084	1.36 (1.26-1.45)	1.35 (75.18)
≥ 7 and < 13	Injection site erythema	2070	3.67 (3.48-3.87)	3.49 (2607.28)
	Erythema	1437	3.43 (3.22-3.65)	3.32 (1661.79)
	Injection site swelling	1377	3.24 (3.04-3.46)	3.14 (1458.37)
	Pyrexia	887	1.03 (0.96-1.10)	1.03 (0.63)
	Infection	865	22.72 (19.93-25.89)	22.09 (4571.52)
	Injection site warmth	849	3.44 (3.17-3.73)	3.37 (999.86)
	Pruritus	784	2.71 (2.50-2.94)	2.66 (615.25)
	Injection site pain	743	2.00 (1.84-2.17)	1.97 (288.34)
	Rash	664	1.62 (1.49-1.77)	1.61 (129.28)
	Skin warm	545	3.32 (3.00-3.67)	3.28 (612.24)

PT, preferred term; a, number of reports containing both the suspect drug and the suspect adverse drug reaction; ROR, reporting odds ratio; CI, confidence interval; PRR, proportional reporting ratio; χ2, Chi-squared.

For children aged 1 to 2 years, the three PTs with the highest reporting counts were pyrexia (a = 7,383; ROR = 0.93; PRR = 0.93), rash (a = 5,146; ROR = 1.90; PRR = 1.84), and infection (a = 2,767; ROR = 5.57; PRR = 5.43). Among those in the 3 to 6 years age range, the top three significant PTs by frequency were injection site erythema (a = 3,836; ROR = 1.23; PRR = 1.21), injection site swelling (a = 2,644; ROR = 1.44; PRR = 1.41), and infection (a = 2,062; ROR = 15.93; PRR = 15.32). For the 7 to 12 years cohort, the predominant significant PTs comprised injection site erythema (a = 2,070; ROR = 3.67; PRR = 3.49), erythema (a = 1,437; ROR = 3.43; PRR = 3.32), and injection site swelling (a = 1,377; ROR = 3.24; PRR = 3.14). **Supplementary Figure S2** illustrates the association between signal strength (ROR) and statistical significance for SOC-level AEs related to varicella vaccination, shown separately for the three age groupings.

### SAEs with survival

Within the VAERS database, SAEs occurred in 5.94% (3,293/55,475) of children aged 1 to 12 years after varicella immunization. Among all SAE cases, pyrexia ranked first in reporting frequency (a = 1,162; ROR = 1.01; PRR = 1.01), followed by convulsion (a = 386; ROR = 1.31; PRR = 1.30) and rash (a = 380; ROR = 1.64; PRR = 1.63). The top 20 positive SAE PT signals for varicella vaccines are illustrated in **Figure 4**, and all identified positive PT signals are provided in **Supplementary Table S6**. Among PTs with both ROR and PRR ≥10, the top 10 PTs ranked by signal strength in descending order were: Varicella zoster virus serology positive (a=16, ROR = 110.74, PRR = 110.66), Varicella post vaccine (a=72, ROR = 99.92, PRR = 99.6), Herpes zoster (a=114, ROR = 37.74, PRR = 37.55), Varicella zoster serology negative (a=5, ROR = 34.59, PRR = 34.58), Varicella virus test positive (a=86, ROR = 28.43, PRR = 28.32), Central nervous system neoplasm (a=4, ROR = 27.67, PRR = 27.67), Cd8 lymphocytes decreased (a=4, ROR = 27.67, PRR = 27.67), Disseminated varicella (a=4, ROR = 27.67, PRR = 27.67), Rash vesicular (a=95, ROR = 22.75, PRR = 22.66), and Ophthalmic herpes zoster (a=6, ROR = 20.75, PRR = 20.75). At the SOC level, the largest proportion of SAEs fell under investigations (a = 6,073; ROR = 1.07; PRR = 1.05), followed by general disorders and administration site conditions (a = 3,117; ROR = 0.79; PRR = 0.82) and nervous system disorders (a = 2,988; ROR = 0.92; PRR = 0.93). The relationship between signal intensity, measured by ROR, and statistical significance for SOC-level SAEs linked to varicella vaccination is depicted in **Supplementary Figure S3A**. Signal strengths for varicella vaccine-related SAEs at the SOC level are summarized in **Supplementary Table S7**.

**Figure 4:**
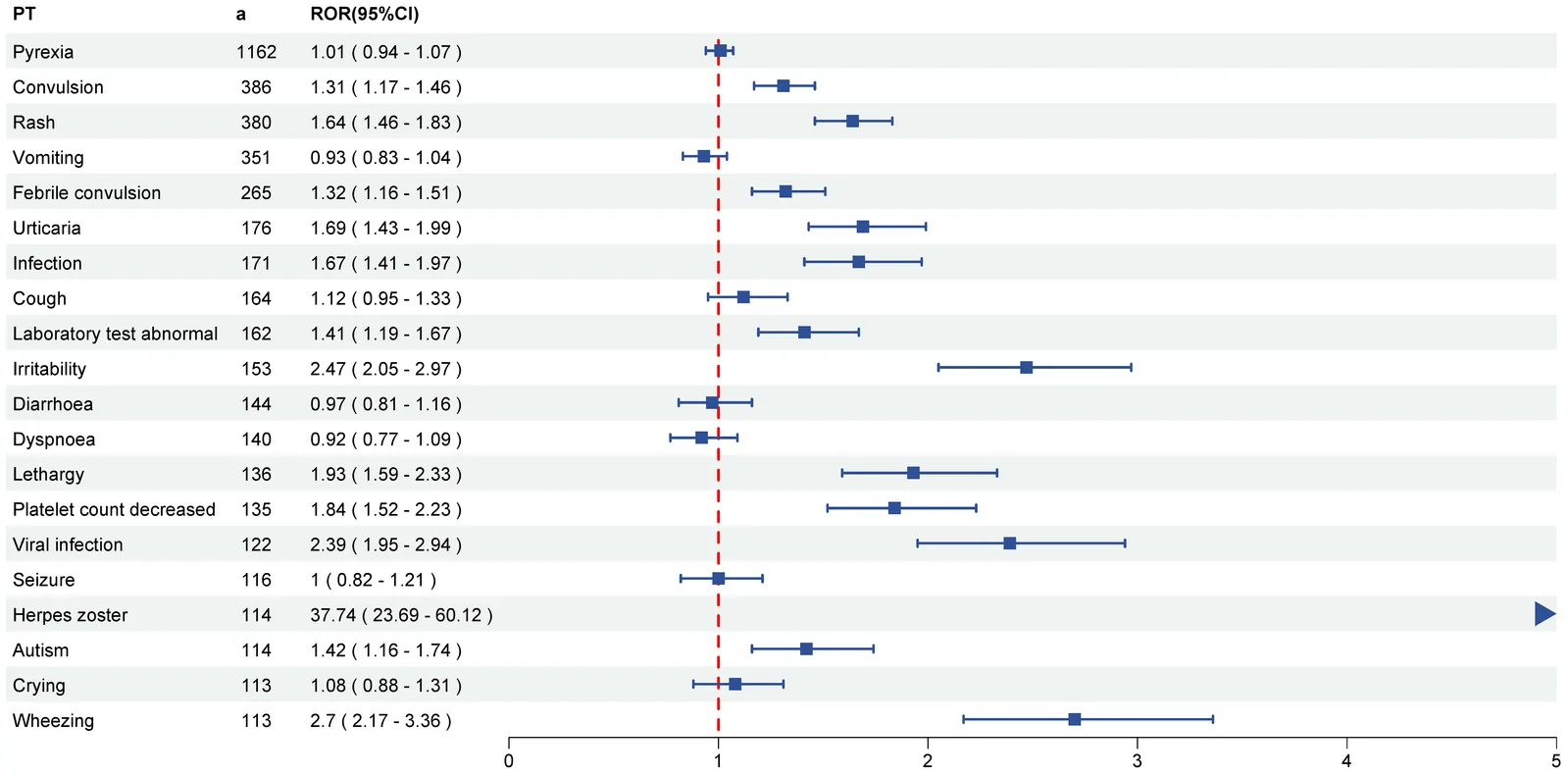
Forest plot of the top twenty positive SAE PT signals associated with varicella vaccines. SAE, serious adverse event; PT, preferred term; ROR, reporting odds ratio.

### SAEs with death

For children aged 1 to 12 years in VAERS, the proportion of death reports after varicella vaccination was 0.30% (165/55,475). At the PT level, death was the most frequently reported death event (a = 82, ROR = 0.83, PRR = 0.84), followed by pyrexia (a = 46, ROR = 0.88, PRR = 0.89) and autopsy (a = 29, ROR = 1.53, PRR = 1.52). The three PTs with stronger signal strength were skin ulcer (a = 4, ROR = 24.32, PRR = 24.25), pupillary reflex impaired (a = 3, ROR = 18.22, PRR = 18.18), and rash papular (a = 5, ROR = 15.21, PRR = 15.15) (**Table 4**). At the SOC level, the investigations had the highest number of deaths (a = 346, ROR = 1.35, PRR = 1.26), then general disorders and administration site conditions (a = 209, ROR = 0.78, PRR = 0.82), and infections and infestations (a = 133, ROR = 0.89, PRR = 0.90). The association between signal strength (ROR) and statistical significance for these SOC-level death-related SAEs is displayed in **Supplementary Figure S3B**, and **Supplementary Table S8** presents their signal strengths.

**Table 4:** Signal strength of varicella-related fatal SAE positive PT signals and their corresponding SOCs.

SOC	PT	a	ROR (95%Cl)	PRR (χ^2^)	Age (years), mean (range)
Nervous system disorders	Unresponsive to stimuli	12	2.09 (1.08-4.03)	2.08 (5.02)	2.16 (1.00-5.00)
General disorders and administration site conditions	Sudden infant death syndrome	10	2.25 (1.09-4.67)	2.24 (5.06)	1.16 (1.01-1.40)
Nervous system disorders	Lethargy	10	2.17 (1.05-4.49)	2.16 (4.64)	2.09 (1.01–5.00)
Respiratory, thoracic and mediastinal disorders	Respiratory distress	9	3.22 (1.43-7.25)	3.21 (8.97)	2.81 (1.00-11.00)
Investigations	Chest x-ray abnormal	7	3.28 (1.30-8.23)	3.26 (7.16)	2.18 (1.00-6.00)
Investigations	Blood culture negative	6	4.05 (1.44-11.41)	4.04 (8.25)	2.22 (1.01-4.00)
Investigations	Pulse absent	5	3.80 (1.24-11.63)	3.79 (6.32)	1.40 (1.00-3.00)
Vascular disorders	Hypertension	5	5.07 (1.54-16.63)	5.05 (8.87)	2.70 (1.00-9.00)
General disorders and administration site conditions	Crying	5	3.04 (1.04-8.90)	3.03 (4.54)	1.67 (1.00-2.00)
Renal and urinary disorders	Renal failure	5	3.04 (1.04-8.90)	3.03 (4.54)	2.82 (1.00-9.00)
Skin and subcutaneous tissue disorders	Rash papular	5	15.21 (2.95-78.47)	15.15 (18.9)	1.18 (1.05-1.50)
Blood and lymphatic system disorders	Coagulopathy	4	4.05 (1.14-14.37)	4.04 (5.50)	2.75 (1.00-7.00)
Skin and subcutaneous tissue disorders	Skin ulcer	4	24.32 (2.72-217.73)	24.25 (17.84)	3.16 (1.05-9.00)
Immune system disorders	Immune system disorder	4	4.86 (1.30-18.13)	4.85 (6.80)	1.51 (1.00-3.00)
Psychiatric disorders	Mental status changes	4	4.86 (1.30-18.13)	4.85 (6.80)	3.75 (1.01-6.00)
Investigations	Computerised tomogram abnormal	4	4.86 (1.30-18.13)	4.85 (6.80)	1.94 (1.01-4.00)
Skin and subcutaneous tissue disorders	Skin lesion	3	4.55 (1.02-20.37)	4.55 (4.75)	1.26 (1.10-1.50)
Eye disorders	Pupillary reflex impaired	3	18.22 (1.89-175.33)	18.18 (12.18)	3.00 (1.01-4.00)
Nervous system disorders	Epilepsy	3	6.07 (1.22-30.12)	6.06 (6.34)	4.33 (4.00-5.00)

SAE, serious adverse event; SOC, system organ class; PT, preferred term; a, number of reports containing both the suspect drug and the suspect adverse drug reaction; ROR, reporting odds ratio; CI, confidence interval; PRR, proportional reporting ratio; χ2, Chi-squared.

### Sensitivity analysis

In clinical practice, some individuals receive the monovalent varicella vaccine together with other vaccines, such as MMR and hepatitis A, to improve vaccination coverage and reduce the number of injections. To lessen the influence of simultaneous vaccinations on our findings, a sensitivity analysis was carried out. Among all the data included in this study, 19,409 children received the monovalent varicella vaccine alone, accounting for 34.99% of the total, while 36,066 children received it in combination with other vaccines, accounting for 65.01%. The commonly co-administered vaccines are shown in **Supplementary Table S9**; the top three most frequently co-administered vaccines were MMR II, HAVRIX, and IPOL. After excluding co-administration with other vaccines, we identified a total of 90 positive signals, as detailed in **Supplementary Table S10**. Similar to the previous analysis, the most common PTs were Infection (a = 4041, ROR = 30.17, PRR = 27.19), Dermatitis bullous (a = 2619, ROR = 130.85, PRR = 124.3), Rash (a = 2381, ROR = 2.59, PRR = 2.51), Viral infection (a = 1369, ROR = 19.50, PRR = 19.02), and Pruritus (a = 1205, ROR = 2.82, PRR = 2.77) (**Figure 5**).

**Figure 5:**
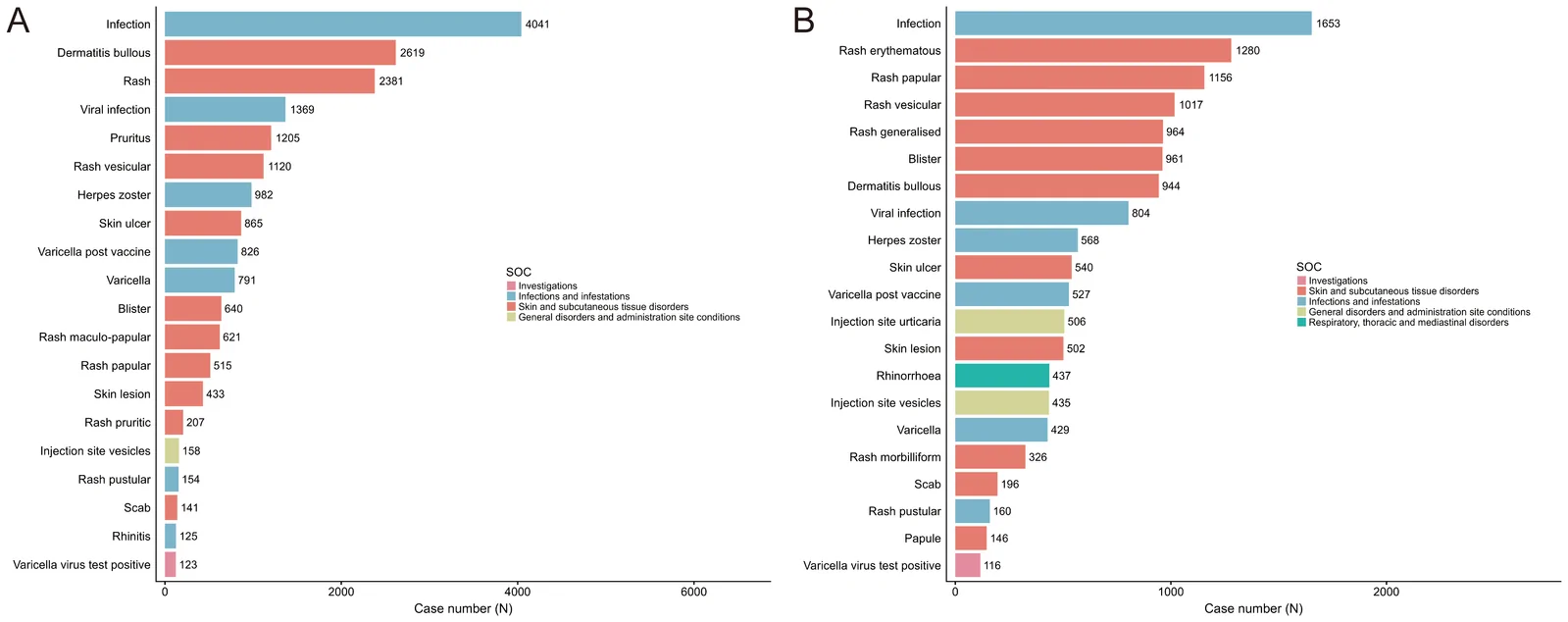
The results of the sensitivity analysis. **(A)** Results of the sensitivity analysis. **(A)** Monovalent varicella vaccine alone. **(B)** Monovalent varicella vaccine combined with other vaccines. SOC, system organ class.

## Discussion

This updated pediatric-focused VAERS disproportionality analysis examined 55,475 AE reports following monovalent varicella vaccination in children aged 1 to 12 years over a 32-year period (1995–2026). It provides recent pharmacovigilance evidence for this population and adds to previous VAERS-based analyses by offering updated signal estimates and age- and sex-specific reporting patterns. This study adds updated pediatric-specific disproportionality results for monovalent varicella vaccines over a 32-year period. Unlike previous all-age VAERS analyses, it focuses on children aged 1–12 years and provides age- and sex-stratified signals. A sensitivity analysis accounting for concomitant vaccination further strengthens the interpretation of the findings. The most frequently reported PTs were rash, infection, dermatitis bullous, viral infection and rash vesicular, and most cases (53.72%) ended in complete recovery. Disproportionality analysis identified expected reactogenicity events, such as injection site reactions and pyrexia, while also revealing distinct patterns across age, sex, and severity strata. Notably, children aged 1 to 2 years accounted for over half of all reports (52.00%), and more than half of the events (52.39%) occurred within the first 6 days after vaccination. SAEs were reported in 5.94% of cases and deaths in 0.30%, with the highest frequencies observed in the SOC of investigations. These findings provide updated real-world evidence that can inform clinical vigilance and regulatory signal assessment for varicella vaccines in the pediatric population.

Our findings are broadly consistent with previous post-licensure surveillance reports on varicella vaccines in children. Known labelled reactions, such as rash, injection site reactions, and fever, were among the most frequently reported adverse events in prior VAERS analyses, with over 95% of reports being non-serious (13, 15). We observed a similar pattern for these known reactions, with rash and injection site reactions ranking among the top events. In contrast, the disproportionality signal for “infection” was notably stronger in our dataset than in earlier studies. This finding should be interpreted as a newly detected signal rather than an established labelled reaction, and it may partly reflect reporting biases, such as misinterpretation of serological tests as vaccine failure, as previously cautioned (15). The proportion of serious reports (5.94%) and deaths (0.30%) in our cohort was slightly higher than the rates of approximately 4% and 0.1% reported in earlier VAERS periods (13, 15); this difference is likely explained by our longer observation window (1995–2026), which includes early years with heightened reporting after licensure, rather than indicating a change in vaccine safety. Finally, vaccination errors, which are non-clinical product-use terms rather than clinical adverse events, accounted for a substantial proportion of reports in recent years (13) but were not separately characterized in our descriptive analysis; this represents a difference in focus rather than a conflicting finding.

Age- and sex-stratified patterns in our study align well with prior evidence. The predominance of reports in children aged 1–2 years and the shift toward local injection site reactions in older children (3–6 and 7–12 years) mirror the age-dependent profiles described for the measles, mumps, rubella and varicella (MMRV) vaccine, where systemic reactions are more common in younger recipients and local reactions in older ones (26). The near-equal sex distribution in our pediatric sample is also consistent with earlier observations that sex differences are minimal in children but emerge in adolescents and adults (14). Methodologically, our application of the Benjamini-Hochberg correction to control false discovery rates reduced the number of positive signals from 135 to 111. This reduction underscores the importance of multiplicity adjustment in pharmacovigilance signal detection and suggests that some rare events may generate false-positive signals due to infrequent reporting, an issue that has not been systematically addressed in previous VAERS studies (13). Our corrected signal set thus provides a more conservative basis for future validation. Compared with a Korean cohort study that found very few vaccine-related events and no VAERS reports after the second dose (14), our results underscore the much higher sensitivity of the U.S. passive surveillance system, though they also highlight the need for caution when interpreting signals because of potential confounding by concomitant vaccinations, as illustrated by Henoch-Schonlein purpura cases reported after co-administration with measles, mumps, rubella (MMR) vaccine (14). A recent review of SAEs after varicella vaccination around the world found similar SAEs in children from the countries of Greece, Germany, Switzerland and Japan (27). Overall, our updated analysis reinforces the established safety profile while offering new granularity on age-specific and methodologically refined signals that warrant further investigation in active surveillance settings.

Our findings carry several implications for pharmacovigilance practice and future health policy. First, this study reinforces the value of VAERS as an early warning system for detecting potential vaccine safety signals (16, 28). Although passive surveillance systems have inherent limitations, including underreporting, reporting bias, and variable data quality, their national scope and timeliness make them indispensable for ongoing vaccine safety monitoring (16). Our systematic disproportionality analysis with multiplicity adjustment provides updated real-world evidence that can assist regulatory agencies and policymakers in continuously evaluating the benefit-risk profile of varicella vaccines. Second, the distinct patterns observed across age and sex subgroups highlight the importance of conducting stratified analyses in future pharmacovigilance studies to identify high-risk populations and develop targeted risk communication strategies (29). Third, our methodological approach, particularly the application of Benjamini-Hochberg correction, offers a more rigorous statistical framework for future VAERS-based research and helps reduce false-positive signals, allowing resources to be focused on truly concerning events (29). Finally, this study underscores the critical need for continued and transparent vaccine safety surveillance to maintain public confidence in immunization programs (30). In an era of growing vaccine hesitancy, evidence-based communication derived from high-quality monitoring data is essential to sustain the success of vaccination policies.

Several limitations of this study should be acknowledged. First, VAERS is a passive surveillance system, and all analyses were based on spontaneously submitted reports. This introduces inherent biases, including underreporting, stimulated reporting, inconsistent data quality, and incomplete clinical information, all of which preclude the calculation of true incidence rates and limit causal inference (31). Second, the lack of reliable denominator data on the number of vaccinated children prevents us from estimating precise reporting rates, and the observed reporting frequencies may be influenced by changes in reporting patterns over time, such as the Weber effect (13, 15). Third, although we applied disproportionality analysis to detect safety signals, these methods are exploratory and cannot establish causal relationships between vaccination and reported adverse events. Thirdly, despite the large overall sample size, some subgroup analyses, particularly for rare events such as deaths and specific serious adverse events, were limited by small numbers, which may affect the stability of signal estimates (32) Finally, it should be noted that MedDRA coding includes not only clinical adverse events but also non-clinical or product-related terms, such as “drug ineffective,” “no adverse event,” and product storage or administration errors. Although we have revised the presentation of the results, these terms remain part of the complete MedDRA-coded dataset. These terms should not be interpreted as true clinical adverse events, and the reported frequencies of clinical adverse events should therefore be interpreted with this distinction in mind. This limitation is inherent to spontaneous reporting data and does not affect the transparency or reproducibility of our analyses. Therefore, all signals identified in this study should be considered hypothesis-generating and require confirmation through well-designed epidemiological studies using active surveillance or other robust methodologies.

## Conclusion

Analysis of 55,475 VAERS reports found that the most frequent adverse events were rash, infection, dermatitis bullous, viral infection and rash vesicular; 53.72% resolved completely. Children aged 1–2 years accounted for 52.00% of reports. Disproportionality analysis identified 135 signals, reduced to 111 after correction. Older children showed more local reactions, while younger children had more systemic events; sex differences were minimal. SAEs occurred in 5.94% and deaths in 0.30%. Herpes zoster was one of the most common SAEs (ROR = 37.74). These findings describe the VAERS reporting profile and support continued pharmacovigilance to inform evidence-based vaccination policies and targeted risk communication.

## Data Availability

Publicly available datasets were analyzed in this study. This data can be found here: The data utilized in these analyses are publicly accessible through the VAERS dataset, available at https://vaers.hhs.gov/index.html.
