# Supplementary material for: Reported adverse events following monovalent varicella vaccination in children aged 1–12 years: a VAERS disproportionality analysis, 1995-2026
Source: Front Immunol. 2026 Oct 29;17:1959638. doi: 10.3389/fimmu.2026.1959638 (PMC13755258; doi:10.3389/fimmu.2026.1959638)
Supplement: Supplementary file 1 [file DataSheet1.zip › supplementary material/supplementary material.docx]

Catalogue

[Table S1. Two-by-two contingency table for measure of disproportionality	2](#_Toc242611116)

[Table S2. Measure of disproportionality and signal generation criteria	3](#_Toc242611117)

[Table S3. Signal strength of all varicella vaccine-related positive PT signals and their corresponding SOCs	4](#_Toc242611118)

[Table S4. Signal strength of AE reports of varicella vaccines at the SOC level.	10](#_Toc242611119)

[Figure S1. Volcano plot of SOCs corresponding to reported AEs in females (A) and males (B).	11](#_Toc242611120)

[Figure S2. Volcano plot of SOCs corresponding to reported AEs in different age groups.	12](#_Toc242611121)

[Table S5. Signal strength of reports of varicella vaccines at the PT level grouped by sex.	13](#_Toc242611122)

[Table S6. Signal strength of varicella vaccine-related positive SAE PT signals and their corresponding SOCs.	14](#_Toc242611123)

[Figure S3. Volcano plot of SOCs corresponding to reported SAEs (A) and deaths (B).	22](#_Toc242611124)

[Table S7. Signal strength of SAE reports of varicella vaccines at the SOC level.	23](#_Toc242611125)

[Table S8. Signal strength of death reports of varicella vaccines at the SOC level.	24](#_Toc242611126)

[Table S9. Summary of all co-administered vaccines.	25](#_Toc242611127)

[Table S10. Signal strength of positive AE PT signals following administration of monovalent varicella vaccine alone and their corresponding SOCs.	28](#_Toc242611128)

# Table S1. Two-by-two contingency table for measure of disproportionality

|  | No. target adverse reaction reports | No. other adverse reaction reports | Total |
| --- | --- | --- | --- |
| Target drug | a | b | a + b |
| Other drugs | c | d | c + d |
| Total | a + c | b + d | N = a + b + c + d |

a, number of reports containing both the suspect drug and the suspect adverse drug reaction; b, number of reports containing the suspect adverse drug reaction with other medications (except the drug of interest); c, number of reports containing the suspect drug with other adverse drug reactions (except the event of interest); d, number of reports containing other medications and other adverse drug reactions.

# Table S2. Measure of disproportionality and signal generation criteria

| Method | Formula | Threshold |
| --- | --- | --- |
| ROR | $ROR=\frac{a/c}{b/d}=\frac{ad}{bc}$  $SE(lnROR)=\sqrt{(\frac{1}{a}+\frac{1}{b}+\frac{1}{c}+\frac{1}{d})}$  $95\% CI=e^{ln\left( ROR \right)\pm1.96\sqrt{(\frac{1}{a}+\frac{1}{b}+\frac{1}{c}+\frac{1}{d})}}$ | a ≥ 3 and 95% CI (lower limit) > 1 |
| PRR | $PRR=\frac{a/{(a+b)}}{c/{(c+d)}}$  $95\% CI=e^{ln(PRR)\pm1.96\sqrt{(\frac{1}{a}-\frac{1}{a+b}+\frac{1}{c}-\frac{1}{c+d})}}$  $\chi^{2}=\frac{{N(\left\vert ad-bc \right\vert-\frac{N}{2})}^{2}}{(a+b)(c+d)(b+d)(a+c)}$ | a ≥ 3 and PRR ≥ 2 and χ^2^ ≥ 4 |

ROR, reporting odds ratio; CI, confidence interval; PRR, proportional reporting ratio; χ^2^, Chi-squared.

# Table S3. Signal strength of all varicella vaccine-related positive PT signals and their corresponding SOCs

| SOC | PT | a | ROR (95%CI) | PRR (χ^2^) | *p*-adjust |
| --- | --- | --- | --- | --- | --- |
| Skin and subcutaneous tissue disorders | Rash | 7218 | 2.09 (2.03-2.15) | 2.05 (2622.99) | ＜0.05 |
| Infections and infestations | Infection | 5694 | 10.76 (10.24-11.31) | 10.43 (13626.36) | ＜0.05 |
| General disorders and administration site conditions | Drug ineffective | 5239 | 19.64 (18.4-20.96) | 19.06 (15722.04) | ＜0.05 |
| Skin and subcutaneous tissue disorders | Dermatitis bullous | 3563 | 37.28 (33.58-41.38) | 36.51 (12252.93) | ＜0.05 |
| Infections and infestations | Viral infection | 2173 | 9.67 (8.95-10.45) | 9.56 (4945.13) | ＜0.05 |
| Skin and subcutaneous tissue disorders | Rash vesicular | 2137 | 20.94 (18.84-23.26) | 20.68 (6528.83) | ＜0.05 |
| Skin and subcutaneous tissue disorders | Rash papular | 1671 | 5.68 (5.27-6.12) | 5.63 (2658.30) | ＜0.05 |
| Skin and subcutaneous tissue disorders | Rash maculo-papular | 1605 | 2.78 (2.61-2.96) | 2.76 (1073.16) | ＜0.05 |
| Skin and subcutaneous tissue disorders | Blister | 1601 | 7.50 (6.90-8.14) | 7.43 (3136.26) | ＜0.05 |
| Skin and subcutaneous tissue disorders | Rash erythematous | 1589 | 2.44 (2.29-2.60) | 2.43 (834.97) | ＜0.05 |
| Infections and infestations | Herpes zoster | 1550 | 24.06 (21.10-27.44) | 23.85 (4901.34) | ＜0.05 |
| Skin and subcutaneous tissue disorders | Skin ulcer | 1405 | 15.93 (14.18-17.90) | 15.81 (3956.64) | ＜0.05 |
| Infections and infestations | Varicella post vaccine | 1353 | 43.47 (36.21-52.18) | 43.13 (4751.71) | ＜0.05 |
| Infections and infestations | Varicella | 1220 | 20.64 (17.97-23.72) | 20.50 (3713.81) | ＜0.05 |
| General disorders and administration site conditions | Injection site rash | 1144 | 2.07 (1.93-2.22) | 2.06 (414.32) | ＜0.05 |
| Skin and subcutaneous tissue disorders | Rash generalised | 1129 | 2.94 (2.72-3.17) | 2.93 (830.46) | ＜0.05 |
| Skin and subcutaneous tissue disorders | Skin lesion | 935 | 7.12 (6.4-7.92) | 7.09 (1771.05) | ＜0.05 |
| Injury, poisoning and procedural complications | Incorrect product storage | 678 | 2.18 (1.98-2.39) | 2.17 (279.31) | ＜0.05 |
| General disorders and administration site conditions | Injection site urticaria | 615 | 2.24 (2.03-2.47) | 2.24 (270.85) | ＜0.05 |
| General disorders and administration site conditions | Injection site vesicles | 593 | 3.59 (3.21-4.01) | 3.58 (584.16) | ＜0.05 |
| Skin and subcutaneous tissue disorders | Rash pruritic | 585 | 2.01 (1.82-2.22) | 2.01 (198.26) | ＜0.05 |
| Skin and subcutaneous tissue disorders | Scab | 337 | 8.86 (7.32-10.73) | 8.85 (732.76) | ＜0.05 |
| Skin and subcutaneous tissue disorders | Rash morbilliform | 333 | 2.11 (1.85-2.41) | 2.11 (127.07) | ＜0.05 |
| Infections and infestations | Rash pustular | 314 | 9.02 (7.39-11.01) | 9.01 (689.71) | ＜0.05 |
| Investigations | Varicella virus test positive | 239 | 14.79 (11.24-19.45) | 14.77 (656.17) | ＜0.05 |
| Skin and subcutaneous tissue disorders | Papule | 178 | 4.59 (3.70-5.69) | 4.58 (232.96) | ＜0.05 |
| General disorders and administration site conditions | Injection site papule | 127 | 5.21 (4.00-6.78) | 5.20 (187.96) | ＜0.05 |
| Investigations | Polymerase chain reaction positive | 78 | 2.37 (1.79-3.14) | 2.37 (38.97) | ＜0.05 |
| Injury, poisoning and procedural complications | Exposure to communicable disease | 75 | 7.53 (5.13-11.06) | 7.53 (147.76) | ＜0.05 |
| Skin and subcutaneous tissue disorders | Acne | 74 | 3.38 (2.48-4.60) | 3.38 (67.28) | ＜0.05 |
| Nervous system disorders | Cerebellar ataxia | 66 | 3.12 (2.26-4.30) | 3.12 (53.48) | ＜0.05 |
| Investigations | Antibody test negative | 65 | 2.33 (1.72-3.17) | 2.33 (31.26) | ＜0.05 |
| Investigations | Culture urine | 63 | 2.43 (1.78-3.33) | 2.43 (33.12) | ＜0.05 |
| Infections and infestations | Viral rash | 59 | 2.76 (1.98-3.84) | 2.76 (39.14) | ＜0.05 |
| Surgical and medical procedures | Cold compress therapy | 55 | 2.87 (2.03-4.06) | 2.87 (39.07) | ＜0.05 |
| Infections and infestations | Infection transmission via personal contact | 52 | 16.07 (8.75-29.51) | 16.07 (146.94) | ＜0.05 |
| Investigations | Viral test | 51 | 2.97 (2.07-4.26) | 2.97 (38.29) | ＜0.05 |
| Investigations | Culture | 49 | 2.03 (1.44-2.86) | 2.03 (16.99) | ＜0.05 |
| Investigations | Varicella virus test negative | 44 | 2.68 (1.83-3.92) | 2.68 (27.75) | ＜0.05 |
| Eye disorders | Eye discharge | 43 | 2.13 (1.47-3.09) | 2.13 (16.89) | ＜0.05 |
| Skin and subcutaneous tissue disorders | Macule | 42 | 3.01 (2.02-4.50) | 3.01 (32.27) | ＜0.05 |
| Investigations | Varicella virus test | 40 | 6.99 (4.18-11.67) | 6.99 (74.89) | ＜0.05 |
| General disorders and administration site conditions | Injection site discharge | 39 | 2.15 (1.45-3.17) | 2.15 (15.55) | ＜0.05 |
| General disorders and administration site conditions | Injection site scab | 37 | 3.91 (2.49-6.15) | 3.91 (40.62) | ＜0.05 |
| Skin and subcutaneous tissue disorders | Dermatitis diaper | 37 | 2.04 (1.37-3.02) | 2.04 (12.94) | ＜0.05 |
| Investigations | Varicella zoster virus serology positive | 37 | 21.23 (9.47-47.63) | 21.23 (113.48) | ＜0.05 |
| Infections and infestations | Roseola | 36 | 3.08 (1.99-4.75) | 3.08 (28.57) | ＜0.05 |
| Infections and infestations | Staphylococcal infection | 36 | 2.16 (1.44-3.24) | 2.16 (14.55) | ＜0.05 |
| Infections and infestations | Injection site pustule | 36 | 4.38 (2.73-7.03) | 4.38 (44.94) | ＜0.05 |
| Infections and infestations | Herpes simplex | 34 | 3.59 (2.26-5.71) | 3.59 (33.59) | ＜0.05 |
| Injury, poisoning and procedural complications | Scar | 34 | 3.69 (2.32-5.88) | 3.69 (34.76) | ＜0.05 |
| Investigations | Blood pressure | 34 | 3.04 (1.94-4.74) | 3.03 (26.42) | ＜0.05 |
| Injury, poisoning and procedural complications | Excoriation | 31 | 3.37 (2.09-5.42) | 3.37 (28.04) | ＜0.05 |
| Skin and subcutaneous tissue disorders | Dermatitis contact | 30 | 2.01 (1.30-3.11) | 2.01 (10.12) | ＜0.05 |
| Injury, poisoning and procedural complications | Arthropod bite | 27 | 3.29 (1.98-5.47) | 3.29 (23.62) | ＜0.05 |
| Infections and infestations | Hand-foot-and-mouth disease | 27 | 2.78 (1.70-4.54) | 2.78 (18.19) | ＜0.05 |
| Infections and infestations | Secondary transmission | 25 | 4.37 (2.48-7.69) | 4.37 (31.08) | ＜0.05 |
| Gastrointestinal disorders | Teething | 24 | 5.36 (2.91-9.87) | 5.36 (36.43) | ＜0.05 |
| General disorders and administration site conditions | Injection site macule | 24 | 2.05 (1.25-3.35) | 2.05 (8.56) | ＜0.05 |
| Gastrointestinal disorders | Oral mucosal eruption | 24 | 2.01 (1.23-3.28) | 2.01 (8.10) | ＜0.05 |
| Infections and infestations | Varicella zoster virus infection | 23 | 11.55 (5.17-25.82) | 11.55 (57.18) | ＜0.05 |
| Ear and labyrinth disorders | Auricular swelling | 22 | 3.68 (2.06-6.57) | 3.68 (22.42) | ＜0.05 |
| Infections and infestations | Impetigo | 22 | 2.21 (1.31-3.72) | 2.21 (9.39) | ＜0.05 |
| Investigations | Varicella zoster serology negative | 20 | 26.78 (7.96-90.12) | 26.78 (64.73) | ＜0.05 |
| Investigations | Viral dna test positive | 18 | 72.30 (9.65-541.63) | 72.3 (66.61) | ＜0.05 |
| Investigations | Measles antibody | 17 | 2.13 (1.18-3.84) | 2.13 (6.69) | ＜0.05 |
| Metabolism and nutrition disorders | Fluid retention | 16 | 2.14 (1.17-3.93) | 2.14 (6.35) | ＜0.05 |
| Infections and infestations | Furuncle | 15 | 2.51 (1.32-4.79) | 2.51 (8.39) | ＜0.05 |
| Infections and infestations | Candidiasis | 14 | 3.75 (1.81-7.77) | 3.75 (14.59) | ＜0.05 |
| Investigations | Culture wound negative | 14 | 4.33 (2.03-9.2) | 4.33 (17.23) | ＜0.05 |
| Skin and subcutaneous tissue disorders | Skin necrosis | 13 | 3.26 (1.57-6.78) | 3.26 (11.26) | ＜0.05 |
| Skin and subcutaneous tissue disorders | Dermatitis acneiform | 13 | 3.07 (1.49-6.32) | 3.07 (10.29) | ＜0.05 |
| Reproductive system and breast disorders | Genital rash | 13 | 2.27 (1.15-4.48) | 2.27 (5.90) | ＜0.05 |
| Infections and infestations | Ophthalmic herpes zoster | 12 | 16.07 (4.53-56.94) | 16.07 (33.91) | ＜0.05 |
| Investigations | Blood lead | 12 | 2.30 (1.13-4.67) | 2.30 (5.58) | ＜0.05 |
| Gastrointestinal disorders | Aphthous stomatitis | 11 | 2.45 (1.16-5.2) | 2.45 (5.88) | ＜0.05 |
| Surgical and medical procedures | Bladder catheterisation | 11 | 3.16 (1.43-6.95) | 3.16 (9.07) | ＜0.05 |
| General disorders and administration site conditions | Vaccination site vesicles | 11 | 3.16 (1.43-6.95) | 3.16 (9.07) | ＜0.05 |
| Social circumstances | Sick relative | 11 | 3.40 (1.52-7.59) | 3.40 (10.09) | ＜0.05 |
| Infections and infestations | Folliculitis | 10 | 4.46 (1.81-10.98) | 4.46 (12.73) | ＜0.05 |
| Surgical and medical procedures | Ear tube insertion | 10 | 2.36 (1.08-5.16) | 2.36 (4.95) | 0.05 |
| Investigations | Drug screen | 10 | 3.65 (1.55-8.60) | 3.65 (10.08) | ＜0.05 |
| Investigations | Virus culture | 9 | 5.16 (1.92-13.87) | 5.16 (13.22) | ＜0.05 |
| Surgical and medical procedures | Heat therapy | 9 | 2.78 (1.19-6.51) | 2.78 (6.06) | ＜0.05 |
| Skin and subcutaneous tissue disorders | Exfoliative rash | 9 | 2.41 (1.05-5.51) | 2.41 (4.64) | 0.07 |
| Infections and infestations | Viral skin infection | 9 | 12.05 (3.26-44.51) | 12.05 (22.80) | ＜0.05 |
| Infections and infestations | Superinfection | 8 | 5.36 (1.86-15.44) | 5.36 (12.14) | ＜0.05 |
| Infections and infestations | Disseminated varicella | 8 | 32.13 (4.02-256.92) | 32.13 (26.81) | ＜0.05 |
| Investigations | Acoustic stimulation tests | 8 | 2.92 (1.17-7.26) | 2.92 (5.85) | ＜0.05 |
| Investigations | Herpes simplex test | 8 | 2.68 (1.09-6.55) | 2.68 (5.05) | 0.06 |
| Eye disorders | Keratitis | 7 | 4.69 (1.57-13.94) | 4.69 (9.37) | ＜0.05 |
| Investigations | Blood lead normal | 7 | 2.81 (1.07-7.39) | 2.81 (4.81) | 0.07 |
| Investigations | Cd4 lymphocytes decreased | 7 | 2.81 (1.07-7.39) | 2.81 (4.81) | 0.07 |
| Social circumstances | Familial risk factor | 7 | 3.12 (1.16-8.39) | 3.12 (5.69) | ＜0.05 |
| Gastrointestinal disorders | Malabsorption | 6 | 3.01 (1.05-8.68) | 3.01 (4.61) | 0.08 |
| Pregnancy, puerperium and perinatal conditions | Premature baby | 6 | 8.03 (2.01-32.12) | 8.03 (12.31) | ＜0.05 |
| Investigations | Aspiration bone marrow abnormal | 6 | 3.44 (1.16-10.24) | 3.44 (5.60) | 0.05 |
| Investigations | Culture wound positive | 6 | 3.01 (1.05-8.68) | 3.01 (4.61) | 0.08 |
| Injury, poisoning and procedural complications | Product name confusion | 5 | 4.02 (1.16-13.87) | 4.02 (5.66) | 0.05 |
| Infections and infestations | Infection parasitic | 5 | 6.69 (1.6-28.01) | 6.69 (9.08) | ＜0.05 |
| Infections and infestations | Abscess bacterial | 5 | 3.35 (1.02-10.97) | 3.35 (4.49) | 0.09 |
| Neoplasms benign, malignant and unspecified (incl cysts and polyps) | Neuroblastoma | 5 | 5.02 (1.35-18.7) | 5.02 (7.15) | ＜0.05 |
| Gastrointestinal disorders | Lip ulceration | 5 | 3.35 (1.02-10.97) | 3.35 (4.49) | 0.09 |
| Blood and lymphatic system disorders | Bone marrow failure | 5 | 3.35 (1.02-10.97) | 3.35 (4.49) | 0.09 |
| Investigations | Herpes simplex serology positive | 5 | 3.35 (1.02-10.97) | 3.35 (4.49) | 0.09 |
| Investigations | Smear site unspecified abnormal | 5 | 6.69 (1.60-28.01) | 6.69 (9.08) | ＜0.05 |
| Investigations | Staphylococcus test negative | 5 | 5.02 (1.35-18.7) | 5.02 (7.15) | ＜0.05 |
| Investigations | Csf virus identified | 5 | 6.69 (1.60-28.01) | 6.69 (9.08) | ＜0.05 |
| Investigations | Viral titre decreased | 5 | 6.69 (1.60-28.01) | 6.69 (9.08) | ＜0.05 |
| Neoplasms benign, malignant and unspecified (incl cysts and polyps) | Central nervous system neoplasm | 4 | 16.07 (1.80-143.75) | 16.07 (11.30) | ＜0.05 |
| Investigations | Csf neutrophil count | 4 | 8.03 (1.47-43.86) | 8.03 (8.21) | ＜0.05 |
| Infections and infestations | Disseminated varicella zoster vaccine virus infection | 4 | 8.03 (1.47-43.86) | 8.03 (8.21) | ＜0.05 |
| Investigations | Cd8 lymphocytes decreased | 4 | 5.36 (1.20-23.93) | 5.36 (6.07) | 0.05 |
| Investigations | Csf white blood cell differential | 4 | 8.03 (1.47-43.86) | 8.03 (8.21) | ＜0.05 |
| Musculoskeletal and connective tissue disorders | Fluctuance | 4 | 5.36 (1.20-23.93) | 5.36 (6.07) | 0.05 |
| Injury, poisoning and procedural complications | Eschar | 4 | 5.36 (1.20-23.93) | 5.36 (6.07) | 0.05 |
| Investigations | Viral dna test negative | 4 | 8.03 (1.47-43.86) | 8.03 (8.21) | ＜0.05 |
| Investigations | B-lymphocyte count decreased | 4 | 8.03 (1.47-43.86) | 8.03 (8.21) | ＜0.05 |
| Infections and infestations | Viraemia | 4 | 16.07 (1.80-143.75) | 16.07 (11.30) | ＜0.05 |
| Investigations | Septic screen | 3 | 6.02 (1.01-36.06) | 6.02 (5.03) | 0.09 |
| Eye disorders | Corneal opacity | 3 | 12.05 (1.25-115.84) | 12.05 (7.60) | ＜0.05 |
| Infections and infestations | Herpes zoster meningoencephalitis | 3 | 12.05 (1.25-115.84) | 12.05 (7.60) | ＜0.05 |
| Skin and subcutaneous tissue disorders | Rosacea | 3 | 12.05 (1.25-115.84) | 12.05 (7.60) | ＜0.05 |
| Investigations | Cell marker | 3 | 6.02 (1.01-36.06) | 6.02 (5.03) | 0.09 |
| Metabolism and nutrition disorders | Diabetes mellitus insulin-dependent | 3 | 6.02 (1.01-36.06) | 6.02 (5.03) | 0.09 |
| Infections and infestations | Herpes zoster disseminated | 3 | 6.02 (1.01-36.06) | 6.02 (5.03) | 0.09 |
| Gastrointestinal disorders | Oral soft tissue disorder | 3 | 12.05 (1.25-115.84) | 12.05 (7.60) | ＜0.05 |
| Skin and subcutaneous tissue disorders | Onychomadesis | 3 | 6.02 (1.01-36.06) | 6.02 (5.03) | 0.09 |
| Investigations | Methicillin-resistant staphylococcal aureus test positive | 3 | 12.05 (1.25-115.84) | 12.05 (7.60) | ＜0.05 |
| General disorders and administration site conditions | Application site vesicles | 3 | 12.05 (1.25-115.84) | 12.05 (7.60) | ＜0.05 |
| Investigations | Tuberculin test positive | 3 | 6.02 (1.01-36.06) | 6.02 (5.03) | 0.09 |
| Infections and infestations | Herpes zoster oticus | 3 | 12.05 (1.25-115.84) | 12.05 (7.60) | ＜0.05 |
| Surgical and medical procedures | Supportive care | 3 | 6.02 (1.01-36.06) | 6.02 (5.03) | 0.09 |
| Respiratory, thoracic and mediastinal disorders | Respiratory tract oedema | 3 | 6.02 (1.01-36.06) | 6.02 (5.03) | 0.09 |
| Investigations | Blood ketone body absent | 3 | 12.05 (1.25-115.84) | 12.05 (7.60) | ＜0.05 |

PT, preferred term; a, number of reports containing both the suspect drug and the suspect adverse drug reaction; ROR, reporting odds ratio; CI, confidence interval; PRR, proportional reporting ratio; χ^2^, Chi-squared.

# Table S4. Signal strength of AE reports of varicella vaccines at the SOC level.

| SOC | a | ROR (95%Cl) | PRR (χ^2^) |
| --- | --- | --- | --- |
| General disorders and administration site conditions | 59651 | 1.10 (1.09-1.12) | 1.07 (303.94) |
| Skin and subcutaneous tissue disorders | 38744 | 2.47 (2.44-2.51) | 2.13 (17575.11) |
| Infections and infestations | 17298 | 2.69 (2.64-2.75) | 2.52 (10273.65) |
| Investigations | 12399 | 0.68 (0.67-0.69) | 0.70 (1464.62) |
| Nervous system disorders | 9340 | 0.50 (0.49-0.51) | 0.53 (3818.45) |
| Injury, poisoning and procedural complications | 7405 | 0.45 (0.44-0.46) | 0.47 (4232.64) |
| Gastrointestinal disorders | 4583 | 0.58 (0.56-0.60) | 0.59 (1185.68) |
| Respiratory, thoracic and mediastinal disorders | 4546 | 0.83 (0.80-0.86) | 0.83 (127.76) |
| Psychiatric disorders | 4056 | 0.75 (0.72-0.77) | 0.75 (289.18) |
| Musculoskeletal and connective tissue disorders | 1848 | 0.42 (0.40-0.44) | 0.42 (1333.08) |
| Vascular disorders | 1659 | 0.50 (0.47-0.53) | 0.50 (732.16) |
| Eye disorders | 1600 | 0.71 (0.68-0.75) | 0.72 (154.14) |
| Metabolism and nutrition disorders | 1310 | 0.79 (0.74-0.84) | 0.79 (62.33) |
| Blood and lymphatic system disorders | 990 | 0.74 (0.69-0.79) | 0.74 (77.61) |
| Immune system disorders | 829 | 0.76 (0.71-0.82) | 0.76 (50.81) |
| Surgical and medical procedures | 620 | 0.73 (0.67-0.80) | 0.73 (51.31) |
| Ear and labyrinth disorders | 276 | 0.58 (0.51-0.65) | 0.58 (75.15) |
| Cardiac disorders | 218 | 0.32 (0.28-0.37) | 0.32 (289.30) |
| Renal and urinary disorders | 160 | 0.39 (0.33-0.46) | 0.39 (141.10) |
| Social circumstances | 119 | 0.34 (0.28-0.40) | 0.34 (144.05) |
| Neoplasms benign, malignant and unspecified (incl cysts and polyps) | 78 | 0.93 (0.72-1.19) | 0.93 (0.37) |
| Reproductive system and breast disorders | 74 | 0.30 (0.24-0.38) | 0.30 (112.23) |
| Hepatobiliary disorders | 68 | 0.44 (0.35-0.57) | 0.44 (42.64) |
| Product issues | 58 | 0.29 (0.22-0.38) | 0.29 (95.39) |
| Congenital, familial and genetic disorders | 50 | 0.62 (0.46-0.83) | 0.62 (10.24) |
| Pregnancy, puerperium and perinatal conditions | 9 | 0.93 (0.45-1.91) | 0.93 (0.04) |
| Endocrine disorders | 9 | 0.18 (0.09-0.35) | 0.18 (32.70) |

AE, adverse event; SOC, System Organ Class; a, number of reports containing both the suspect drug and the suspect adverse drug reaction; ROR, reporting odds ratio; CI, confidence interval; PRR, proportional reporting ratio; χ^2^, Chi-squared.

A: Females	B: Males

# Figure S1. Volcano plot of SOCs corresponding to reported AEs in females (A) and males (B).

SOC, system organ classes; AEs, adverse events; ROR, reporting odds ratio.

A: ≥ 1 and ≤ 2	B: ≥ 3 and ≤ 6	C: ≥ 7 and ≤ 12

# Figure S2. Volcano plot of SOCs corresponding to reported AEs in different age groups.

SOC, system organ classes; AEs, adverse events; ROR, reporting odds ratio.

# Table S5. Signal strength of reports of varicella vaccines at the PT level grouped by sex.

|  | PT | a | ROR (95%CI) | PRR (χ^2^) |
| --- | --- | --- | --- | --- |
| Female | Pyrexia | 4991 | 1.18 (1.14-1.22) | 1.17 (96.35) |
|  | Injection site erythema | 4337 | 1.68 (1.62-1.74) | 1.64 (803.64) |
|  | Rash | 3493 | 2.13 (2.04-2.22) | 2.08 (1322.06) |
|  | Infection | 2658 | 11.37 (10.56-12.24) | 11.02 (6553.19) |
|  | Injection site swelling | 2581 | 1.64 (1.57-1.72) | 1.62 (446.40) |
|  | Erythema | 2252 | 1.66 (1.58-1.74) | 1.64 (406.19) |
|  | Dermatitis bullous | 1739 | 39.43 (33.84-45.94) | 38.58 (6060.51) |
|  | Injection site warmth | 1634 | 1.58 (1.49-1.67) | 1.57 (245.72) |
|  | Pruritus | 1431 | 1.79 (1.68-1.91) | 1.78 (343.21) |
|  | Urticaria | 1254 | 1.01 (0.95-1.07) | 1.01 (0.06) |
| Male | Pyrexia | 5215 | 1.07 (1.04-1.11) | 1.07 (19.05) |
|  | Injection site erythema | 3820 | 1.23 (1.19-1.28) | 1.22 (124.96) |
|  | Rash | 3590 | 2.07 (1.98-2.15) | 2.02 (1263.44) |
|  | Infection | 2899 | 10.71 (9.99-11.48) | 10.37 (6925.10) |
|  | Injection site swelling | 2473 | 1.25 (1.19-1.31) | 1.24 (91.51) |
|  | Erythema | 2149 | 1.27 (1.21-1.33) | 1.26 (91.26) |
|  | Dermatitis bullous | 1771 | 36.18 (31.26-41.87) | 35.43 (6068.03) |
|  | Urticaria | 1551 | 1.11 (1.05-1.18) | 1.11 (13.13) |
|  | Injection site warmth | 1456 | 1.14 (1.08-1.21) | 1.14 (20.19) |
|  | Pruritus | 1402 | 1.63 (1.53-1.73) | 1.61 (236.92) |

PT, preferred term; a, number of reports containing both the suspect drug and the suspect adverse drug reaction; ROR, reporting odds ratio; CI, confidence interval; PRR, proportional reporting ratio; χ^2^, Chi-squared.

# Table S6. Signal strength of varicella vaccine-related positive SAE PT signals and their corresponding SOCs.

| SOC | PT | a | ROR (95%CI) | PRR (χ^2^) |
| --- | --- | --- | --- | --- |
| Psychiatric disorders | Irritability | 153 | 2.47 (2.05-2.97) | 2.46 (98.20) |
| Infections and infestations | Viral infection | 122 | 2.39 (1.95-2.94) | 2.38 (73.07) |
| Infections and infestations | Herpes zoster | 114 | 37.74 (23.69-60.12) | 37.55 (631.32) |
| Respiratory, thoracic and mediastinal disorders | Wheezing | 113 | 2.70 (2.17-3.36) | 2.70 (86.89) |
| Investigations | Full blood count | 100 | 2.29 (1.82-2.87) | 2.28 (54.32) |
| Skin and subcutaneous tissue disorders | Rash vesicular | 95 | 22.75 (15.01-34.49) | 22.66 (460.27) |
| Investigations | Varicella virus test positive | 86 | 28.43 (17.64-45.82) | 28.32 (445.19) |
| Investigations | Full blood count normal | 84 | 2.29 (1.79-2.93) | 2.29 (45.82) |
| Investigations | Polymerase chain reaction | 72 | 3.20 (2.42-4.23) | 3.19 (74.26) |
| Infections and infestations | Varicella post vaccine | 72 | 99.92 (40.36-247.38) | 99.6 (456.55) |
| Injury, poisoning and procedural complications | Contusion | 71 | 2.09 (1.60-2.73) | 2.09 (31.04) |
| Skin and subcutaneous tissue disorders | Blister | 71 | 5.47 (4.01-7.47) | 5.46 (144.59) |
| Skin and subcutaneous tissue disorders | Dermatitis bullous | 69 | 13.29 (8.88-19.9) | 13.26 (268.25) |
| Skin and subcutaneous tissue disorders | Skin lesion | 68 | 6.46 (4.64-8.99) | 6.44 (162.01) |
| Respiratory, thoracic and mediastinal disorders | Hypoxia | 65 | 2.13 (1.62-2.82) | 2.13 (29.86) |
| Surgical and medical procedures | Endotracheal intubation | 62 | 2.41 (1.81-3.22) | 2.41 (37.95) |
| Skin and subcutaneous tissue disorders | Rash erythematous | 53 | 2.42 (1.77-3.3) | 2.41 (32.51) |
| General disorders and administration site conditions | Swelling face | 52 | 2.00 (1.47-2.72) | 2.00 (20.14) |
| Skin and subcutaneous tissue disorders | Skin ulcer | 51 | 10.1 (6.57-15.53) | 10.08 (169.82) |
| Investigations | Metabolic function test | 45 | 2.51 (1.79-3.54) | 2.51 (30.02) |
| Nervous system disorders | Status epilepticus | 43 | 2.01 (1.43-2.83) | 2.01 (16.92) |
| Blood and lymphatic system disorders | Idiopathic thrombocytopenic purpura | 43 | 2.92 (2.04-4.17) | 2.92 (38.11) |
| Psychiatric disorders | Decreased eye contact | 40 | 2.11 (1.48-3.01) | 2.11 (17.96) |
| Investigations | Viral test negative | 38 | 2.19 (1.52-3.16) | 2.19 (18.68) |
| Skin and subcutaneous tissue disorders | Rash papular | 38 | 3.21 (2.18-4.72) | 3.21 (39.42) |
| Nervous system disorders | Cerebellar ataxia | 36 | 3.90 (2.59-5.86) | 3.89 (49.51) |
| Infections and infestations | Varicella | 36 | 9.59 (5.79-15.89) | 9.58 (116.00) |
| Investigations | Culture urine negative | 35 | 2.04 (1.40-2.97) | 2.03 (14.23) |
| Investigations | Metabolic function test normal | 34 | 2.12 (1.44-3.11) | 2.12 (15.38) |
| Investigations | Polymerase chain reaction positive | 34 | 5.89 (3.73-9.30) | 5.88 (74.45) |
| Gastrointestinal disorders | Lip swelling | 34 | 2.59 (1.74-3.84) | 2.58 (24.06) |
| Psychiatric disorders | Social avoidant behaviour | 31 | 2.33 (1.55-3.51) | 2.33 (17.63) |
| General disorders and administration site conditions | Developmental delay | 31 | 2.47 (1.64-3.72) | 2.46 (19.90) |
| Eye disorders | Eye swelling | 30 | 2.93 (1.91-4.48) | 2.92 (26.69) |
| Investigations | Csf glucose normal | 29 | 2.68 (1.74-4.11) | 2.67 (21.94) |
| Skin and subcutaneous tissue disorders | Eczema | 28 | 2.48 (1.61-3.83) | 2.48 (18.26) |
| Skin and subcutaneous tissue disorders | Scab | 27 | 11.00 (5.99-20.18) | 10.99 (94.70) |
| Respiratory, thoracic and mediastinal disorders | Nasal congestion | 26 | 2.12 (1.36-3.29) | 2.12 (11.72) |
| Eye disorders | Eyelid oedema | 25 | 2.25 (1.43-3.53) | 2.25 (13.05) |
| Investigations | Csf white blood cell count increased | 22 | 2.27 (1.40-3.68) | 2.27 (11.79) |
| Metabolism and nutrition disorders | Oral intake reduced | 22 | 3.11 (1.88-5.14) | 3.11 (21.68) |
| Investigations | Respiratory rate increased | 20 | 2.47 (1.48-4.12) | 2.47 (12.90) |
| Psychiatric disorders | Regressive behaviour | 18 | 2.15 (1.27-3.64) | 2.15 (8.42) |
| Investigations | Herpes simplex serology negative | 18 | 3.11 (1.79-5.43) | 3.11 (17.80) |
| Respiratory, thoracic and mediastinal disorders | Sneezing | 17 | 2.61 (1.50-4.57) | 2.61 (12.29) |
| Investigations | Computerised tomogram abnormal | 17 | 2.18 (1.26-3.76) | 2.18 (8.24) |
| Investigations | Viral test | 16 | 2.99 (1.66-5.38) | 2.99 (14.81) |
| Investigations | Varicella zoster virus serology positive | 16 | 110.74 (14.69-835.13) | 110.66 (102.29) |
| Investigations | Differential white blood cell count | 15 | 2.73 (1.50-4.97) | 2.73 (11.80) |
| Investigations | Csf white blood cell count negative | 15 | 2.00 (1.12-3.55) | 2 (5.78) |
| Infections and infestations | Rash pustular | 14 | 4.61 (2.35-9.07) | 4.61 (23.76) |
| General disorders and administration site conditions | Injection site vesicles | 14 | 2.55 (1.38-4.71) | 2.55 (9.63) |
| Investigations | Culture urine | 14 | 2.48 (1.35-4.58) | 2.48 (9.13) |
| Immune system disorders | Immunodeficiency | 14 | 5.70 (2.81-11.56) | 5.7 (29.73) |
| Hepatobiliary disorders | Hepatic function abnormal | 13 | 2.14 (1.15-3.99) | 2.14 (6.04) |
| Investigations | Csf red blood cell count positive | 13 | 3.00 (1.56-5.75) | 3.00 (12.07) |
| Surgical and medical procedures | Transfusion | 13 | 2.57 (1.36-4.86) | 2.57 (9.09) |
| Psychiatric disorders | Head banging | 12 | 2.86 (1.46-5.61) | 2.86 (10.28) |
| Blood and lymphatic system disorders | Pancytopenia | 12 | 2.59 (1.34-5.04) | 2.59 (8.55) |
| Investigations | Varicella virus test | 12 | 9.23 (3.89-21.9) | 9.22 (37.70) |
| Infections and infestations | Respiratory syncytial virus infection | 12 | 2.77 (1.42-5.41) | 2.77 (9.67) |
| Investigations | Blood albumin normal | 12 | 2.24 (1.17-4.3) | 2.24 (6.24) |
| Psychiatric disorders | Stereotypy | 12 | 2.31 (1.20-4.43) | 2.31 (6.66) |
| Investigations | Red blood cells csf positive | 12 | 3.08 (1.56-6.07) | 3.07 (11.63) |
| Infections and infestations | Staphylococcal infection | 11 | 2.82 (1.40-5.68) | 2.82 (9.17) |
| Psychiatric disorders | Attention deficit/hyperactivity disorder | 11 | 2.24 (1.13-4.42) | 2.24 (5.69) |
| Surgical and medical procedures | Chemotherapy | 11 | 3.04 (1.50-6.19) | 3.04 (10.48) |
| Investigations | Lymphocyte count decreased | 11 | 2.31 (1.17-4.56) | 2.31 (6.10) |
| Psychiatric disorders | Childhood disintegrative disorder | 11 | 2.31 (1.17-4.56) | 2.31 (6.10) |
| Infections and infestations | Herpes simplex | 10 | 3.46 (1.62-7.39) | 3.46 (11.65) |
| General disorders and administration site conditions | Sudden infant death syndrome | 10 | 2.56 (1.24-5.29) | 2.56 (6.95) |
| Infections and infestations | Varicella zoster virus infection | 10 | 17.3 (5.42-55.16) | 17.29 (43.86) |
| Infections and infestations | Meningitis aseptic | 10 | 3.14 (1.49-6.64) | 3.14 (10.05) |
| Investigations | Differential white blood cell count normal | 10 | 2.31 (1.13-4.72) | 2.31 (5.55) |
| Renal and urinary disorders | Renal failure | 9 | 2.22 (1.05-4.71) | 2.22 (4.58) |
| Eye disorders | Periorbital oedema | 9 | 2.71 (1.25-5.85) | 2.71 (6.96) |
| Investigations | Culture wound negative | 9 | 12.45 (4.17-37.17) | 12.45 (33.85) |
| Injury, poisoning and procedural complications | Exposure to communicable disease | 9 | 8.90 (3.31-23.89) | 8.89 (27.59) |
| Investigations | Hiv test negative | 9 | 2.39 (1.12-5.11) | 2.39 (5.43) |
| Nervous system disorders | Cerebral infarction | 8 | 3.46 (1.48-8.08) | 3.46 (9.32) |
| Surgical and medical procedures | Incisional drainage | 8 | 2.52 (1.12-5.65) | 2.52 (5.36) |
| Neoplasms benign, malignant and unspecified (incl cysts and polyps) | Acute lymphocytic leukaemia | 8 | 2.77 (1.22-6.28) | 2.77 (6.45) |
| Nervous system disorders | Clumsiness | 8 | 2.52 (1.12-5.65) | 2.52 (5.36) |
| Investigations | Scan with contrast normal | 8 | 3.69 (1.56-8.70) | 3.69 (10.23) |
| Musculoskeletal and connective tissue disorders | Joint effusion | 8 | 2.52 (1.12-5.65) | 2.52 (5.36) |
| Skin and subcutaneous tissue disorders | Pruritus generalised | 8 | 2.77 (1.22-6.28) | 2.77 (6.45) |
| Blood and lymphatic system disorders | Red blood cell abnormality | 7 | 3.73 (1.49-9.34) | 3.72 (9.07) |
| Investigations | Morbillivirus test positive | 7 | 2.69 (1.12-6.44) | 2.69 (5.35) |
| Eye disorders | Eye oedema | 7 | 4.40 (1.71-11.36) | 4.40 (11.25) |
| Injury, poisoning and procedural complications | Scar | 7 | 2.85 (1.18-6.87) | 2.85 (5.95) |
| Investigations | Csf lymphocyte count increased | 7 | 4.40 (1.71-11.36) | 4.40 (11.25) |
| Investigations | Immunology test normal | 7 | 2.42 (1.02-5.73) | 2.42 (4.32) |
| Eye disorders | Eye discharge | 7 | 3.46 (1.40-8.57) | 3.46 (8.16) |
| Respiratory, thoracic and mediastinal disorders | Respiratory depression | 7 | 6.05 (2.19-16.7) | 6.05 (15.75) |
| Infections and infestations | Adenovirus infection | 7 | 2.55 (1.07-6.06) | 2.55 (4.81) |
| Surgical and medical procedures | Oxygen supplementation | 7 | 2.85 (1.18-6.87) | 2.85 (5.95) |
| Investigations | Immunoglobulins | 7 | 4.04 (1.59-10.25) | 4.03 (10.09) |
| Surgical and medical procedures | Ear tube insertion | 7 | 4.84 (1.84-12.72) | 4.84 (12.55) |
| Investigations | Allergy test positive | 7 | 2.42 (1.02-5.73) | 2.42 (4.32) |
| Investigations | Respiratory syncytial virus test positive | 7 | 2.42 (1.02-5.73) | 2.42 (4.32) |
| Investigations | Gram stain | 7 | 4.04 (1.59-10.25) | 4.03 (10.09) |
| Infections and infestations | Pneumonia aspiration | 6 | 2.59 (1.01-6.63) | 2.59 (4.27) |
| Nervous system disorders | Apraxia | 6 | 2.77 (1.07-7.13) | 2.77 (4.84) |
| Gastrointestinal disorders | Malabsorption | 6 | 8.30 (2.53-27.20) | 8.3 (17.51) |
| Psychiatric disorders | Posturing | 6 | 5.19 (1.80-14.96) | 5.19 (11.59) |
| Infections and infestations | Ophthalmic herpes zoster | 6 | 20.75 (4.19-102.84) | 20.75 (28.20) |
| Investigations | Acoustic stimulation tests | 6 | 20.75 (4.19-102.84) | 20.75 (28.20) |
| Nervous system disorders | Encephalitis autoimmune | 6 | 2.96 (1.14-7.72) | 2.96 (5.47) |
| Investigations | International normalised ratio | 6 | 3.19 (1.21-8.40) | 3.19 (6.18) |
| Investigations | Blood immunoglobulin g decreased | 6 | 3.77 (1.40-10.20) | 3.77 (7.91) |
| Investigations | Acoustic stimulation tests normal | 6 | 5.19 (1.80-14.96) | 5.19 (11.59) |
| Ear and labyrinth disorders | Auricular swelling | 6 | 13.84 (3.46-55.33) | 13.83 (23.81) |
| Investigations | Skin test positive | 6 | 2.96 (1.14-7.72) | 2.96 (5.47) |
| Skin and subcutaneous tissue disorders | Skin necrosis | 5 | 4.94 (1.57-15.57) | 4.94 (9.17) |
| Infections and infestations | Candidiasis | 5 | 3.84 (1.29-11.47) | 3.84 (6.76) |
| Investigations | Blood gases abnormal | 5 | 2.88 (1.02-8.18) | 2.88 (4.34) |
| Musculoskeletal and connective tissue disorders | Fasciitis | 5 | 2.88 (1.02-8.18) | 2.88 (4.34) |
| Neoplasms benign, malignant and unspecified (incl cysts and polyps) | Brain neoplasm | 5 | 4.32 (1.41-13.22) | 4.32 (7.86) |
| Skin and subcutaneous tissue disorders | Dermatitis contact | 5 | 8.65 (2.32-32.21) | 8.65 (15.03) |
| Investigations | Cd4 lymphocytes decreased | 5 | 8.65 (2.32-32.21) | 8.65 (15.03) |
| Surgical and medical procedures | Bladder catheterisation | 5 | 4.94 (1.57-15.57) | 4.94 (9.17) |
| Surgical and medical procedures | Platelet transfusion | 5 | 3.84 (1.29-11.47) | 3.84 (6.76) |
| Nervous system disorders | Complex partial seizures | 5 | 3.84 (1.29-11.47) | 3.84 (6.76) |
| Investigations | Csf protein decreased | 5 | 3.84 (1.29-11.47) | 3.84 (6.76) |
| Investigations | Biopsy skin | 5 | 8.65 (2.32-32.21) | 8.65 (15.03) |
| Investigations | Globulin | 5 | 2.88 (1.02-8.18) | 2.88 (4.34) |
| Investigations | Varicella zoster serology negative | 5 | 34.59 (4.04-296.10) | 34.58 (27.18) |
| Investigations | Magnetic resonance imaging brain abnormal | 5 | 2.88 (1.02-8.18) | 2.88 (4.34) |
| Neoplasms benign, malignant and unspecified (incl cysts and polyps) | Central nervous system neoplasm | 4 | 27.67 (3.09-247.59) | 27.67 (20.56) |
| Infections and infestations | Superinfection | 4 | 9.22 (2.06-41.21) | 9.22 (12.57) |
| Investigations | Csf lymphocyte count normal | 4 | 3.46 (1.04-11.49) | 3.46 (4.66) |
| Investigations | Csf neutrophil count | 4 | 13.84 (2.53-75.54) | 13.83 (15.87) |
| Neoplasms benign, malignant and unspecified (incl cysts and polyps) | Neuroblastoma | 4 | 13.84 (2.53-75.54) | 13.83 (15.87) |
| Investigations | Roseolovirus test positive | 4 | 3.46 (1.04-11.49) | 3.46 (4.66) |
| Investigations | Natural killer cell count | 4 | 3.46 (1.04-11.49) | 3.46 (4.66) |
| Gastrointestinal disorders | Oral pain | 4 | 4.61 (1.30-16.34) | 4.61 (6.79) |
| Infections and infestations | Disseminated varicella zoster vaccine virus infection | 4 | 13.84 (2.53-75.54) | 13.83 (15.87) |
| Investigations | Cd8 lymphocytes decreased | 4 | 27.67 (3.09-247.59) | 27.67 (20.56) |
| Infections and infestations | Disseminated varicella | 4 | 27.67 (3.09-247.59) | 27.67 (20.56) |
| Metabolism and nutrition disorders | Fluid retention | 4 | 3.46 (1.04-11.49) | 3.46 (4.66) |
| Nervous system disorders | Infant irritability | 4 | 6.92 (1.73-27.66) | 6.92 (10.12) |
| Investigations | Csf white blood cell count | 4 | 5.53 (1.49-20.61) | 5.53 (8.25) |
| Investigations | Cd8 lymphocytes | 4 | 3.95 (1.16-13.50) | 3.95 (5.61) |
| Eye disorders | Dark circles under eyes | 4 | 4.61 (1.30-16.34) | 4.61 (6.79) |
| Investigations | Albumin globulin ratio normal | 4 | 5.53 (1.49-20.61) | 5.53 (8.25) |
| Investigations | Herpes simplex test | 4 | 3.46 (1.04-11.49) | 3.46 (4.66) |
| Investigations | Drug screen | 4 | 4.61 (1.30-16.34) | 4.61 (6.79) |
| Investigations | Csf white blood cell differential | 4 | 13.84 (2.53-75.54) | 13.83 (15.87) |
| Investigations | Amino acid level | 4 | 3.95 (1.16-13.50) | 3.95 (5.61) |
| Investigations | T-lymphocyte count decreased | 4 | 5.53 (1.49-20.61) | 5.53 (8.25) |
| Investigations | Culture wound | 4 | 13.84 (2.53-75.54) | 13.83 (15.87) |
| Investigations | Borrelia burgdorferi serology negative | 4 | 3.46 (1.04-11.49) | 3.46 (4.66) |
| Investigations | Staphylococcus test negative | 4 | 6.92 (1.73-27.66) | 6.92 (10.12) |
| Investigations | Csf virus identified | 4 | 9.22 (2.06-41.21) | 9.22 (12.57) |
| General disorders and administration site conditions | Injection site discharge | 4 | 3.95 (1.16-13.50) | 3.95 (5.61) |
| Investigations | Aspiration bone marrow abnormal | 4 | 3.95 (1.16-13.50) | 3.95 (5.61) |
| Investigations | Chromosome analysis normal | 4 | 9.22 (2.06-41.21) | 9.22 (12.57) |
| Surgical and medical procedures | Stem cell transplant | 4 | 9.22 (2.06-41.21) | 9.22 (12.57) |
| Cardiac disorders | Cardiac failure congestive | 3 | 5.19 (1.16-23.18) | 5.19 (5.79) |
| Infections and infestations | Abscess bacterial | 3 | 6.92 (1.40-34.28) | 6.92 (7.59) |
| Investigations | B-lymphocyte count | 3 | 5.19 (1.16-23.18) | 5.19 (5.79) |
| Pregnancy, puerperium and perinatal conditions | Premature baby | 3 | 20.75 (2.16-199.52) | 20.75 (14.10) |
| Metabolism and nutrition disorders | Failure to thrive | 3 | 5.19 (1.16-23.18) | 5.19 (5.79) |
| Investigations | Cd4/cd8 ratio | 3 | 6.92 (1.40-34.28) | 6.92 (7.59) |
| Investigations | Fungal test negative | 3 | 5.19 (1.16-23.18) | 5.19 (5.79) |
| Investigations | Audiogram abnormal | 3 | 5.19 (1.16-23.18) | 5.19 (5.79) |
| Metabolism and nutrition disorders | Diabetes mellitus insulin-dependent | 3 | 20.75 (2.16-199.52) | 20.75 (14.10) |
| Investigations | Culture wound positive | 3 | 5.19 (1.16-23.18) | 5.19 (5.79) |
| Respiratory, thoracic and mediastinal disorders | Intercostal retraction | 3 | 10.38 (1.73-62.10) | 10.37 (10.17) |
| Immune system disorders | Atopy | 3 | 10.38 (1.73-62.10) | 10.37 (10.17) |
| Investigations | B-lymphocyte count decreased | 3 | 20.75 (2.16-199.52) | 20.75 (14.10) |
| Investigations | Clostridium test positive | 3 | 5.19 (1.16-23.18) | 5.19 (5.79) |
| Respiratory, thoracic and mediastinal disorders | Respiratory tract oedema | 3 | 20.75 (2.16-199.52) | 20.75 (14.10) |
| Musculoskeletal and connective tissue disorders | Joint contracture | 3 | 6.92 (1.40-34.28) | 6.92 (7.59) |

SAE, serious adverse event; PT, preferred term; SOC, system organ class; a, number of reports containing both the suspect drug and the suspect adverse drug reaction; CI, confidence interval; ROR, reporting odds ratio; PRR, proportional reporting ratio; χ^2^, Chi-squared.

A: SAEs	B: Deaths

# Figure S3. Volcano plot of SOCs corresponding to reported SAEs (A) and deaths (B).

SOC, system organ classes; SAEs, serious adverse events; ROR, reporting odds ratio

# Table S7. Signal strength of SAE reports of varicella vaccines at the SOC level.

| SOC | a | ROR (95%CI) | PRR (χ^2^) |
| --- | --- | --- | --- |
| General disorders and administration site conditions | 59651 | 1.10 (1.09-1.12) | 1.07 (303.94) |
| Skin and subcutaneous tissue disorders | 38744 | 2.47 (2.44-2.51) | 2.13 (17575.11) |
| Infections and infestations | 17298 | 2.69 (2.64-2.75) | 2.52 (10273.65) |
| Investigations | 12399 | 0.68 (0.67-0.69) | 0.70 (1464.62) |
| Nervous system disorders | 9340 | 0.50 (0.49-0.51) | 0.53 (3818.45) |
| Injury, poisoning and procedural complications | 7405 | 0.45 (0.44-0.46) | 0.47 (4232.64) |
| Gastrointestinal disorders | 4583 | 0.58 (0.56-0.60) | 0.59 (1185.68) |
| Respiratory, thoracic and mediastinal disorders | 4546 | 0.83 (0.80-0.86) | 0.83 (127.76) |
| Psychiatric disorders | 4056 | 0.75 (0.72-0.77) | 0.75 (289.18) |
| Musculoskeletal and connective tissue disorders | 1848 | 0.42 (0.40-0.44) | 0.42 (1333.08) |
| Vascular disorders | 1659 | 0.50 (0.47-0.53) | 0.50 (732.16) |
| Eye disorders | 1600 | 0.71 (0.68-0.75) | 0.72 (154.14) |
| Metabolism and nutrition disorders | 1310 | 0.79 (0.74-0.84) | 0.79 (62.33) |
| Blood and lymphatic system disorders | 990 | 0.74 (0.69-0.79) | 0.74 (77.61) |
| Immune system disorders | 829 | 0.76 (0.71-0.82) | 0.76 (50.81) |
| Surgical and medical procedures | 620 | 0.73 (0.67-0.80) | 0.73 (51.31) |
| Ear and labyrinth disorders | 276 | 0.58 (0.51-0.65) | 0.58 (75.15) |
| Cardiac disorders | 218 | 0.32 (0.28-0.37) | 0.32 (289.30) |
| Renal and urinary disorders | 160 | 0.39 (0.33-0.46) | 0.39 (141.10) |
| Social circumstances | 119 | 0.34 (0.28-0.40) | 0.34 (144.05) |
| Neoplasms benign, malignant and unspecified (incl cysts and polyps) | 78 | 0.93 (0.72-1.19) | 0.93 (0.37) |
| Reproductive system and breast disorders | 74 | 0.30 (0.24-0.38) | 0.30 (112.23) |
| Hepatobiliary disorders | 68 | 0.44 (0.35-0.57) | 0.44 (42.64) |
| Product issues | 58 | 0.29 (0.22-0.38) | 0.29 (95.39) |
| Congenital, familial and genetic disorders | 50 | 0.62 (0.46-0.83) | 0.62 (10.24) |
| Pregnancy, puerperium and perinatal conditions | 9 | 0.93 (0.45-1.91) | 0.93 (0.04) |
| Endocrine disorders | 9 | 0.18 (0.09-0.35) | 0.18 (32.70) |

SAE, serious adverse event; SOC, system organ class; a, number of reports containing both the suspect drug and the suspect adverse drug reaction; ROR, reporting odds ratio; CI, confidence interval; PRR, proportional reporting ratio; χ^2^, Chi-squared.

# Table S8. Signal strength of death reports of varicella vaccines at the SOC level.

| SOC | a | ROR (95%CI) | PRR (χ^2^) |
| --- | --- | --- | --- |
| Investigations | 346 | 1.35 (1.18-1.54) | 1.26 (19.25) |
| General disorders and administration site conditions | 209 | 0.78 (0.67-0.92) | 0.82 (9.09) |
| Infections and infestations | 133 | 0.89 (0.73-1.07) | 0.90 (1.55) |
| Nervous system disorders | 128 | 0.81 (0.67-0.98) | 0.83 (4.54) |
| Respiratory, thoracic and mediastinal disorders | 102 | 1.04 (0.84-1.30) | 1.04 (0.15) |
| Gastrointestinal disorders | 53 | 0.78 (0.58-1.05) | 0.79 (2.77) |
| Cardiac disorders | 45 | 0.93 (0.67-1.28) | 0.93 (0.21) |
| Surgical and medical procedures | 41 | 0.94 (0.67-1.31) | 0.94 (0.13) |
| Skin and subcutaneous tissue disorders | 39 | 1.71 (1.19-2.45) | 1.69 (8.73) |
| Blood and lymphatic system disorders | 38 | 1.49 (1.04-2.14) | 1.48 (4.80) |
| Metabolism and nutrition disorders | 36 | 1.77 (1.21-2.57) | 1.75 (9.06) |
| Vascular disorders | 29 | 0.95 (0.64-1.42) | 0.96 (0.05) |
| Psychiatric disorders | 27 | 0.96 (0.64-1.45) | 0.96 (0.03) |
| Injury, poisoning and procedural complications | 16 | 0.93 (0.55-1.58) | 0.93 (0.07) |
| Hepatobiliary disorders | 15 | 1.45 (0.82-2.55) | 1.44 (1.66) |
| Neoplasms benign, malignant and unspecified (incl cysts and polyps) | 14 | 2.03 (1.11-3.73) | 2.02 (5.44) |
| Eye disorders | 11 | 0.95 (0.50-1.80) | 0.95 (0.02) |
| Immune system disorders | 10 | 0.75 (0.39-1.44) | 0.75 (0.76) |
| Renal and urinary disorders | 10 | 1.19 (0.60-2.35) | 1.19 (0.25) |
| Congenital, familial and genetic disorders | 7 | 1.18 (0.52-2.66) | 1.18 (0.16) |
| Musculoskeletal and connective tissue disorders | 6 | 0.36 (0.16-0.83) | 0.37 (6.23) |
| Reproductive system and breast disorders | 1 | 0.61 (0.08-4.74) | 0.61 (0.23) |
| Endocrine disorders | 1 | 0.36 (0.05-2.68) | 0.36 (1.10) |
| Pregnancy, puerperium and perinatal conditions | 1 | 3.03 (0.27-33.47) | 3.03 (0.91) |
| Ear and labyrinth disorders | 1 | 0.26 (0.04-1.95) | 0.26 (1.98) |
| Social circumstances | 1 | 0.38 (0.05-2.86) | 0.38 (0.96) |

SOC, system organ class; a, number of reports containing both the suspect drug and the suspect adverse drug reaction; ROR, reporting odds ratio; CI, confidence interval; PRR, proportional reporting ratio; χ^2^, Chi-squared.

# Table S9. Summary of all co-administered vaccines.

| Vaccine type | Vaccine name | N |
| --- | --- | --- |
| MMR | MMR II | 22210 |
| HEPA | HAVRIX | 5804 |
| IPV | IPOL | 4648 |
| PNC | PREVNAR | 3805 |
| DTAP | INFANRIX | 3211 |
| PNC13 | PREVNAR13 | 3176 |
| DTAP | DAPTACEL | 2972 |
| HEPA | VAQTA | 2864 |
| DTAPIPV | KINRIX | 2771 |
| HIBV | ACTHIB | 2252 |
| FLU3 | SEASONAL | 2072 |
| DTAP | TRIPEDIA | 1759 |
| MNQ | MENACTRA | 1626 |
| HIBV | PEDVAXHIB | 1569 |
| TDAP | ADACEL | 1254 |
| MMRV | PROQUAD | 1234 |
| TDAP | BOOSTRIX | 1142 |
| HBHEPB | COMVAX | 974 |
| IPV | POLIOVAX | 846 |
| HPV4 | GARDASIL | 842 |
| HEP | ENGERIX-B | 819 |
| FLU4 | SEASONAL | 731 |
| HEP | RECOMBIVAX HB | 727 |
| DTAPIPVHIB | PENTACEL | 689 |
| DTAPHEPBIP | PEDIARIX | 666 |
| OPV | ORIMUNE | 659 |
| HIBV | HIBTITER | 567 |
| MMR | VIRIVAC | 494 |
| DTAP | ACEL-IMUNE | 298 |
| FLUN3 | SEASONAL | 291 |
| MMR | PRIORIX | 239 |
| PNC20 | PREVNAR20 | 233 |
| MNQ | MENVEO | 186 |
| DTAPIPV | QUADRACEL | 178 |
| MMR | MMR I | 163 |
| DTPHIB | TETRAMUNE | 161 |
| HIBV | PROHIBIT | 139 |
| HPV9 | GARDASIL 9 | 122 |
| FLU(H1N1) | H1N1 | 121 |
| DTAPH | TRIHIBIT | 118 |
| PPV | PNEUMOVAX | 115 |
| HIBV | HIBERIX | 113 |
| PNC15 | VAXNEUVANCE | 101 |
| HEP | FOREIGN | 85 |
| PPV | PNU-IMUNE | 57 |
| COVID19 | MODERNA | 51 |
| FLUN4 | SEASONAL | 50 |
| COVID19 | PFIZER-BIONTECH | 48 |
| MNQ | MENQUADFI | 45 |
| DTPPVHBHPB | VAXELIS | 44 |
| MEN | MENOMUNE | 42 |
| HBPV | HIBIMUNE | 41 |
| UNK | OTHER | 39 |
| VARZOS | ZOSTAVAX | 39 |
| DTP | TRI-IMMUNOL | 38 |
| FLUN(H1N1) | H1N1 | 31 |
| MER | MR-VAX II | 31 |
| MENB | BEXSERO | 27 |
| FLUC4 | SEASONAL | 22 |
| MM | MM-VAX | 22 |
| COVID19-2 | PFIZER-BIONTECH BIVALENT | 18 |
| TD | TENIVAC | 18 |
| DTAPIPV | UNKNOWN | 17 |
| DTPHIB | DTP + ACTHIB | 14 |
| TYP | TYPHIM VI | 14 |
| FLUX(H1N1) | H1N1 | 13 |
| HEPAB | TWINRIX | 13 |
| MEA | ATTENUVAX | 13 |
| RV5 | ROTATEQ | 13 |
| PNC10 | SYNFLORIX | 12 |
| TD | TDVAX | 12 |
| COVID19-2 | MODERNA BIVALENT | 11 |
| DTAP | CERTIVA | 11 |
| FLUX | SEASONAL | 11 |
| DTAPIPV | INFANRIX TETRA | 9 |
| DTAPIPVHIB | UNKNOWN | 8 |
| HIBV | OMNIHIB | 8 |
| HPV2 | CERVARIX | 8 |
| HEP | GENHEVAC B | 7 |
| MENB | TRUMENBA | 6 |
| DT | DECAVAC | 5 |
| DT | DITANRIX | 5 |
| FLUA4 | SEASONAL | 5 |
| MNC | MENINGITEC | 5 |
| MUR | FOREIGN | 5 |
| PNC21 | CAPVAXIVE | 5 |
| 6VAX-F | HEXAVAC | 4 |
| 6VAX-F | INFANRIX HEXA | 4 |
| DTAPIPVHIB | INFANRIX QUINTA | 4 |
| FLUA3 | SEASONAL | 4 |
| HEP | HEPLISAV-B | 4 |
| RAB | IMOVAX | 4 |
| FLUC3 | SEASONAL | 3 |
| RV1 | ROTARIX | 3 |
| BCG | MYCOBAX | 2 |
| CHOL | USP | 2 |
| DPP | QUATRO VIRELON | 2 |
| JEV | JE-VAX | 2 |
| MEN | MENOMUNE-A/C | 2 |
| MENHIB | MENITORIX | 2 |
| MNQHIB | MENHIBRIX | 2 |
| MU | MUMPSVAX II | 2 |
| RAB | RABAVERT | 2 |
| RV | ROTASHIELD | 2 |
| TDAPIPV | FOREIGN | 2 |
| YF | YF-VAX | 2 |
| DF | DENGVAXIA | 1 |
| DTPHEP | TRITANRIX | 1 |
| FLUR3 | SEASONAL | 1 |
| FLUR4 | SEASONAL | 1 |
| MENHIB | UNKNOWN | 1 |
| RUB | MERUVAX II | 1 |
| SMALL | ACAM2000 | 1 |
| SMALL | DRYVAX | 1 |
| TBE | TICOVAC | 1 |
| TD | DITANRIX | 1 |
| TTOX | TEVAX | 1 |
| UNK | FOREIGN | 1 |
| VARZOS | SHINGRIX | 1 |
| YF | STAMARIL | 1 |

# Table S10. Signal strength of positive AE PT signals following administration of monovalent varicella vaccine alone and their corresponding SOCs.

| SOC | PT | a | ROR (95%Cl) | PRR (χ^2^) |
| --- | --- | --- | --- | --- |
| Infections and infestations | Infection | 4041 | 30.17 (28.24-32.23) | 27.9 (23557.05) |
| Skin and subcutaneous tissue disorders | Dermatitis bullous | 2619 | 130.85 (111.92-152.98) | 124.3 (19425.55) |
| Skin and subcutaneous tissue disorders | Rash | 2381 | 2.59 (2.47-2.71) | 2.51 (1687.26) |
| Infections and infestations | Viral infection | 1369 | 19.50 (17.69-21.51) | 19.02 (6932.34) |
| Skin and subcutaneous tissue disorders | Pruritus | 1205 | 2.82 (2.64-3.01) | 2.77 (1024.82) |
| Skin and subcutaneous tissue disorders | Rash vesicular | 1120 | 44.50 (38.33-51.66) | 43.56 (7226.63) |
| Infections and infestations | Herpes zoster | 982 | 47.21 (40.09-55.59) | 46.33 (6410.90) |
| Skin and subcutaneous tissue disorders | Skin ulcer | 865 | 42.24 (35.76-49.88) | 41.55 (5523.63) |
| Infections and infestations | Varicella post vaccine | 826 | 117.40 (89.75-153.57) | 115.55 (6066.29) |
| Infections and infestations | Varicella | 791 | 49.63 (41.19-59.80) | 48.89 (5213.43) |
| Skin and subcutaneous tissue disorders | Blister | 640 | 12.35 (10.91-13.97) | 12.21 (2607.84) |
| Skin and subcutaneous tissue disorders | Rash maculo-papular | 621 | 4.86 (4.40-5.37) | 4.81 (1173.67) |
| Injury, poisoning and procedural complications | Incorrect product storage | 577 | 3.85 (3.48-4.25) | 3.82 (814.65) |
| Skin and subcutaneous tissue disorders | Rash papular | 515 | 6.32 (5.63-7.10) | 6.27 (1280.08) |
| Skin and subcutaneous tissue disorders | Skin lesion | 433 | 12.00 (10.34-13.92) | 11.91 (1737.63) |
| Skin and subcutaneous tissue disorders | Rash pruritic | 207 | 2.24 (1.92-2.61) | 2.23 (110.38) |
| General disorders and administration site conditions | Injection site vesicles | 158 | 4.48 (3.68-5.44) | 4.47 (272.79) |
| Infections and infestations | Rash pustular | 154 | 15.99 (12.16-21.03) | 15.95 (719.52) |
| Skin and subcutaneous tissue disorders | Scab | 141 | 13.58 (10.35-17.81) | 13.55 (607.34) |
| Infections and infestations | Rhinitis | 125 | 2.05 (1.68-2.49) | 2.05 (53.31) |
| Investigations | Varicella virus test positive | 123 | 23.4 (16.49-33.23) | 23.35 (670.06) |
| Respiratory, thoracic and mediastinal disorders | Pharyngolaryngeal pain | 79 | 3.32 (2.55-4.32) | 3.32 (90.28) |
| Investigations | Antibody test negative | 52 | 4.46 (3.18-6.27) | 4.46 (89.50) |
| Injury, poisoning and procedural complications | Exposure to communicable disease | 52 | 13.39 (8.58-20.89) | 13.37 (222.41) |
| Infections and infestations | Infection transmission via personal contact | 35 | 23.27 (12.08-44.83) | 23.26 (190.35) |
| Investigations | Varicella zoster virus serology positive | 34 | 54.25 (21.22-138.73) | 54.22 (227.72) |
| Investigations | Polymerase chain reaction positive | 33 | 2.41 (1.64-3.57) | 2.41 (20.99) |
| Skin and subcutaneous tissue disorders | Papule | 32 | 3.11 (2.07-4.68) | 3.11 (32.99) |
| Skin and subcutaneous tissue disorders | Acne | 26 | 3.58 (2.25-5.68) | 3.57 (33.29) |
| Investigations | Varicella virus test negative | 26 | 5.06 (3.09-8.27) | 5.06 (51.78) |
| Infections and infestations | Herpes simplex | 24 | 6.84 (3.96-11.79) | 6.83 (64.37) |
| Nervous system disorders | Cerebellar ataxia | 21 | 3.22 (1.94-5.35) | 3.22 (22.90) |
| Surgical and medical procedures | Cold compress therapy | 21 | 7.98 (4.36-14.61) | 7.97 (64.04) |
| General disorders and administration site conditions | Injection site papule | 21 | 3.81 (2.26-6.40) | 3.81 (29.41) |
| Injury, poisoning and procedural complications | Scar | 20 | 6.14 (3.42-10.99) | 6.13 (48.57) |
| Infections and infestations | Varicella zoster virus infection | 19 | 25.26 (10.09-63.25) | 25.25 (106.20) |
| Respiratory, thoracic and mediastinal disorders | Lung disorder | 18 | 2.28 (1.35-3.85) | 2.28 (10.04) |
| Investigations | Varicella virus test | 17 | 11.30 (5.40-23.66) | 11.30 (66.02) |
| Investigations | Viral test | 17 | 3.48 (1.97-6.15) | 3.48 (20.88) |
| Infections and infestations | Secondary transmission | 16 | 6.38 (3.31-12.31) | 6.38 (40.32) |
| Investigations | Varicella zoster serology negative | 15 | 39.88 (11.54-137.76) | 39.87 (94.74) |
| Investigations | Herpes simplex test negative | 14 | 2.48 (1.36-4.52) | 2.48 (9.44) |
| Skin and subcutaneous tissue disorders | Dermatitis contact | 13 | 3.05 (1.61-5.78) | 3.05 (12.95) |
| Investigations | Viral test positive | 13 | 3.14 (1.65-5.97) | 3.14 (13.61) |
| General disorders and administration site conditions | Injection site scab | 11 | 5.48 (2.54-11.81) | 5.48 (23.89) |
| Infections and infestations | Injection site pustule | 11 | 3.81 (1.86-7.82) | 3.81 (15.45) |
| Gastrointestinal disorders | Oral pain | 10 | 2.28 (1.13-4.60) | 2.28 (5.58) |
| Surgical and medical procedures | Chemotherapy | 10 | 2.57 (1.26-5.25) | 2.57 (7.27) |
| Investigations | Herpes simplex serology negative | 10 | 2.10 (1.05-4.21) | 2.1 (4.55) |
| Infections and infestations | Ophthalmic herpes zoster | 9 | 23.92 (6.48-88.38) | 23.92 (49.42) |
| Infections and infestations | Oral herpes | 9 | 2.11 (1.01-4.40) | 2.11 (4.16) |
| Infections and infestations | Folliculitis | 8 | 21.27 (5.64-80.16) | 21.26 (42.13) |
| Infections and infestations | Roseola | 8 | 2.77 (1.24-6.20) | 2.77 (6.73) |
| Infections and infestations | Hand-foot-and-mouth disease | 8 | 2.28 (1.04-5.00) | 2.28 (4.46) |
| Neoplasms benign, malignant and unspecified (incl cysts and polyps) | Acute lymphocytic leukaemia | 8 | 2.77 (1.24-6.2) | 2.77 (6.73) |
| Metabolism and nutrition disorders | Fluid retention | 8 | 3.99 (1.71-9.32) | 3.99 (11.93) |
| Injury, poisoning and procedural complications | Excoriation | 8 | 3.75 (1.62-8.70) | 3.75 (10.98) |
| Infections and infestations | Impetigo | 8 | 2.36 (1.07-5.20) | 2.36 (4.85) |
| Investigations | Viral dna test positive | 8 | 63.8 (7.98-510.11) | 63.79 (54.94) |
| Infections and infestations | Skin infection | 8 | 3.54 (1.54-8.15) | 3.54 (10.11) |
| Neoplasms benign, malignant and unspecified (incl cysts and polyps) | Leukaemia | 7 | 2.66 (1.13-6.25) | 2.66 (5.43) |
| Skin and subcutaneous tissue disorders | Skin necrosis | 7 | 5.07 (1.97-13.09) | 5.07 (13.99) |
| Reproductive system and breast disorders | Genital rash | 7 | 3.99 (1.61-9.88) | 3.99 (10.44) |
| General disorders and administration site conditions | Mucous membrane disorder | 6 | 3.42 (1.31-8.89) | 3.42 (7.18) |
| Nervous system disorders | Cerebral infarction | 6 | 2.52 (1.01-6.31) | 2.52 (4.17) |
| Infections and infestations | Superinfection | 6 | 7.97 (2.57-24.73) | 7.97 (18.30) |
| Infections and infestations | Disseminated varicella | 6 | 47.85 (5.76-397.45) | 47.84 (39.31) |
| Infections and infestations | Meningitis aseptic | 6 | 2.52 (1.01-6.31) | 2.52 (4.17) |
| Eye disorders | Keratitis | 6 | 7.97 (2.57-24.73) | 7.97 (18.30) |
| Investigations | Csf lymphocyte count increased | 5 | 6.65 (2.03-21.77) | 6.64 (13.08) |
| Investigations | Cd4 lymphocytes decreased | 5 | 4.43 (1.48-13.22) | 4.43 (8.54) |
| Blood and lymphatic system disorders | Bone marrow failure | 5 | 7.97 (2.31-27.55) | 7.97 (15.25) |
| General disorders and administration site conditions | Vaccination site vesicles | 5 | 4.98 (1.63-15.24) | 4.98 (9.80) |
| Investigations | Biopsy skin | 5 | 3.07 (1.09-8.60) | 3.07 (5.03) |
| Investigations | Viral titre decreased | 5 | 19.94 (3.87-102.76) | 19.93 (25.69) |
| Infections and infestations | Infection parasitic | 4 | 15.95 (2.92-87.08) | 15.95 (18.68) |
| Musculoskeletal and connective tissue disorders | Fasciitis | 4 | 3.54 (1.09-11.51) | 3.54 (5.06) |
| Investigations | Csf pressure increased | 4 | 6.38 (1.71-23.76) | 6.38 (10.08) |
| Investigations | Aspiration bone marrow abnormal | 4 | 7.97 (1.99-31.89) | 7.97 (12.20) |
| Investigations | Herpes simplex test | 4 | 4.56 (1.33-15.57) | 4.56 (7.07) |
| Gastrointestinal disorders | Lip ulceration | 3 | 4.78 (1.14-20.02) | 4.78 (5.61) |
| Surgical and medical procedures | Bone marrow transplant | 3 | 5.98 (1.34-26.72) | 5.98 (7.11) |
| Respiratory, thoracic and mediastinal disorders | Nasal oedema | 3 | 4.78 (1.14-20.02) | 4.78 (5.61) |
| Investigations | Seroconversion test negative | 3 | 7.97 (1.61-39.51) | 7.97 (9.15) |
| Investigations | Herpes simplex serology positive | 3 | 4.78 (1.14-20.02) | 4.78 (5.61) |
| Investigations | Viral dna test negative | 3 | 11.96 (2.00-71.59) | 11.96 (12.05) |
| Investigations | Smear site unspecified abnormal | 3 | 11.96 (2.00-71.59) | 11.96 (12.05) |
| Investigations | Csf virus identified | 3 | 7.97 (1.61-39.51) | 7.97 (9.15) |
| Investigations | Biopsy brain abnormal | 3 | 4.78 (1.14-20.02) | 4.78 (5.61) |
| Investigations | Blood immunoglobulin g abnormal | 3 | 4.78 (1.14-20.02) | 4.78 (5.61) |

AE serious adverse event; PT, preferred term; SOC, System Organ Class; a, number of reports containing both the suspect drug and the suspect adverse drug reaction; CI, confidence interval; ROR, reporting odds ratio; PRR, proportional reporting ratio; χ^2^, Chi-squared.
